# A component of the TOR (Target Of Rapamycin) nutrient-sensing pathway plays a role in circadian rhythmicity in *Neurospora crassa*

**DOI:** 10.1371/journal.pgen.1007457

**Published:** 2018-06-20

**Authors:** Lalanthi Ratnayake, Keyur K. Adhvaryu, Elizabeth Kafes, Kamyar Motavaze, Patricia Lakin-Thomas

**Affiliations:** Department of Biology, York University, Toronto, ON, Canada; Oregon State University, UNITED STATES

## Abstract

The TOR (Target of Rapamycin) pathway is a highly-conserved signaling pathway in eukaryotes that regulates cellular growth and stress responses. The cellular response to amino acids or carbon sources such as glucose requires anchoring of the TOR kinase complex to the lysosomal/vacuolar membrane by the Ragulator (mammals) or EGO (yeast) protein complex. Here we report a connection between the TOR pathway and circadian (daily) rhythmicity. The molecular mechanism of circadian rhythmicity in all eukaryotes has long been thought to be transcription/translation feedback loops (TTFLs). In the model eukaryote *Neurospora crassa*, a TTFL including FRQ (frequency) and WCC (white collar complex) has been intensively studied. However, it is also well-known that rhythmicity can be seen in the absence of TTFL functioning. We previously isolated *uv90* as a mutation that compromises FRQ-less rhythms and also damps the circadian oscillator when FRQ is present. We have now mapped the *uv90* gene and identified it as NCU05950, homologous to the TOR pathway proteins EGO1 (yeast) and LAMTOR1 (mammals), and we have named the *N*. *crassa* protein VTA (vacuolar TOR-associated protein). The protein is anchored to the outer vacuolar membrane and deletion of putative acylation sites destroys this localization as well as the protein’s function in rhythmicity. A deletion of VTA is compromised in its growth responses to amino acids and glucose. We conclude that a key protein in the complex that anchors TOR to the vacuole plays a role in maintaining circadian (daily) rhythmicity. Our results establish a connection between the TOR pathway and circadian rhythms and point towards a network integrating metabolism and the circadian system.

## Author summary

Circadian clocks drive 24-hour rhythms in living things at all levels of organization, from single cells to whole organisms. In spite of the importance of daily clocks for organizing the activities and internal functions of organisms, there are still many unsolved problems concerning the molecular mechanisms. In eukaryotes, a set of “clock proteins” turns on and off specific genes in a 24-hour feedback loop. This “clock gene feedback loop” has been the dominant idea about how clocks work for many years. However, some rhythms can still be seen when these feedback loops are not functioning. Using the fungus *Neurospora crassa* as a model organism, we have discovered a gene that is important for maintaining rhythms that continue without the known feedback loop. We have found that this gene codes for a protein that was already known to be important in helping cells to adjust their growth rate to adapt to varying availability of nutrients. Because the same gene is found in all eukaryotes, including mammals, this finding may point towards a universal clock mechanism that integrates nutritional needs with daily rhythms.

## Introduction

The TOR (Target of Rapamycin) pathway is a highly conserved cellular pathway in eukaryotes that monitors nutritional and stress signals from both extracellular and intracellular sources to regulate cellular growth, division, autophagy and stress responses. It has been extensively studied in mammals [1] and yeast [2]. In mammals, extracellular growth factors act through cell-surface receptors to activate the kinase AKT, which in turn activates the mammalian TOR complex 1 (mTORC1) through inactivation of the inhibitory tuberous sclerosis complex (TSC) [3]. The mTORC1 complex is also activated by amino acids through pathways independent of cell-surface receptors and TSC [1]. Monomeric GTPases in the RAG family are localized to the lysosomal membrane by interaction with the membrane-bound RAGULATOR complex. RAGs are activated by amino acids through various mechanisms and recruit mTORC1 to the lysosome. This pathway converges with the growth factor pathway to help activate mTORC1 at the lysosome [3]. In yeast, which do not utilize growth factors and do not have TSC complexes, the primary signals are amino acids and other nutrients such as carbon and nitrogen sources. The RAG-family GTPases Gtr1 and Gtr2 are anchored at the vacuole (homologous to the mammalian lysosome) by binding to the membrane-localized EGO complex (similar to the mammalian RAGULATOR complex). TORC1 is found at the yeast vacuolar membrane regardless of nutrient status, and is activated by amino acids through activation of Gtr1 and Gtr2 [3]. In yeast, there is evidence that other pathways related to amino acid availability also intersect with TORC1 signaling, including the GAAC (general amino acid control) pathway, which upregulates amino acid transport and metabolism in response to amino acid starvation, and the SPS (Ssy1-Ptr3-Ssy5) pathway that upregulates amino acid transport in response to extracellular amino acids [3]. In both yeast and mammals, localization of the TOR complex to the lysosomal/vacuolar membrane is essential for its activation [4]. The key process that leads to increased cell growth is activation of ribosome biogenesis and increased protein translation, accomplished by the phosphorylation of key targets by the activated TORC1 kinase [2, 5, 6]. In mammals, the best-characterized targets are S6K, which in turn phosphorylates ribosomal protein S6 to increase ribosome biogenesis; and translation inhibitor 4E-BP, phosphorylation of which increases translation [2, 5, 6]. In yeast, Sch9 is the S6K homolog, activating ribosome biogenesis, and the second major target is Tap42, a regulator of type 2A phosphatases (PP2A). The two major proximal TORC1 effectors Tap42 and Sch9 together activate translation initiation factors. Disruptions in the TOR pathway have been associated with human pathologies such as metabolic disease, cancer, and age-related diseases [7, 8].

Circadian (daily) rhythmicity is also common to all eukaryotes and some prokaryotes, and rhythmicity is found in many and possibly most intracellular functions including metabolism, stress responses, cell division and growth [9]. Circadian disruption has been linked to multiple disorders in mammals and humans including metabolic syndrome [9, 10]. We have now identified a connection between circadian rhythmicity and the TOR pathway in the model eukaryote *Neurospora crassa*.

The most popular model for the molecular mechanism of rhythmicity in all organisms has been a transcription/translation feedback loop (TTFL) involving the activation of transcription of a gene by a positive element, the translation of that gene into a protein that is the negative element, and the negative feedback of that protein onto the positive element to inhibit transcription. In *N*. *crassa*, the protein product FRQ of the frequency (*frq*) gene is the negative element and the White Collar Complex (WCC) is the positive element [11–13]. Many problems with this model have been noted, not least of which are examples of circadian rhythmicity in the absence of transcription that would be required for a TTFL mechanism [9, 14, 15]. In *N*. *crassa*, which has a long history as a model organism for investigating the molecular mechanisms of circadian rhythmicity, there are numerous examples of rhythmicity in the absence of a TTFL in mutant strains lacking functional FRQ or WCC genes [12]. FRQ-less rhythms of conidiation (spore-formation) have been reported in strains growing on agar medium for long periods of time [16], in cultures exposed to temperature cycles [17–19], in strains with defects in lipid metabolism [20], in cultures supplemented with various chemicals [21, 22], and in a variety of mutant backgrounds [23–26]. FRQ-less rhythms at the molecular level have been reported in the level of diacylglycerol [27], the level of *ccg-16* mRNA [28, 29], the activity of nitrate reductase [30], the level of WC-1 protein [29], and the oxidation state of peroxiredoxin protein [31]. There is very little information about the molecular mechanisms of FRQ-less rhythmicity. A recent report [26] described a mutation, *cog-1*, that allows the expression of rhythms in strains defective in the TTFL, and this rhythmicity required a functional blue light photoreceptor CRY (Cryptochrome) gene, suggesting a role for CRY in rhythmicity.

In order to identify potential components of a FRQ-less circadian oscillator (FLO) [32], we carried out a mutagenesis screen in a FRQ-less strain using UV light. We identified a mutation, named *uv90*, that compromises FRQ-less rhythmicity of the conidiation (spore-formation) rhythm under two different assay systems [33]: rhythmic entrainment to short heat pulses, and free-running rhythmicity in *chol-1* (choline-requiring) strains deprived of choline. Crucially, in the FRQ wild-type background, this mutation also dampens the rhythm of conidiation, dampens the amplitude of the FRQ protein rhythm, and dampens the amplitude of the entire circadian oscillator as measured by responses to phase-resetting by light or heat pulses [33]. The *uv90* mutation therefore identifies a gene that is necessary for robust FRQ-less rhythmicity and also for maintaining the amplitude of the entire circadian system when FRQ is present.

Our goal in the present work was to identify the gene affected by the *uv90* mutation and uncover its functional role. We have mapped and identified the *uv90* gene and have found that it codes for a protein in the TOR pathway that is homologous to p18/LAMTOR1 in mammals and EGO1 in yeast. In yeast and mammals, this protein is essential for the vacuolar/lysosomal localization of the TORC1 complex and participates in the transmission of nutritional status information. We demonstrate that the UV90 protein is localized to the vacuolar membrane, and is required for normal sensing of both amino acids and glucose in the growth medium. Our results contribute to understanding the close connection between metabolic status and circadian rhythmicity by identifying the function of a component of an oscillatory network that can drive rhythmicity independent of the standard TTFL.

## Results

### The *uv90* gene is identified as NCU05950

**Fig 1:**
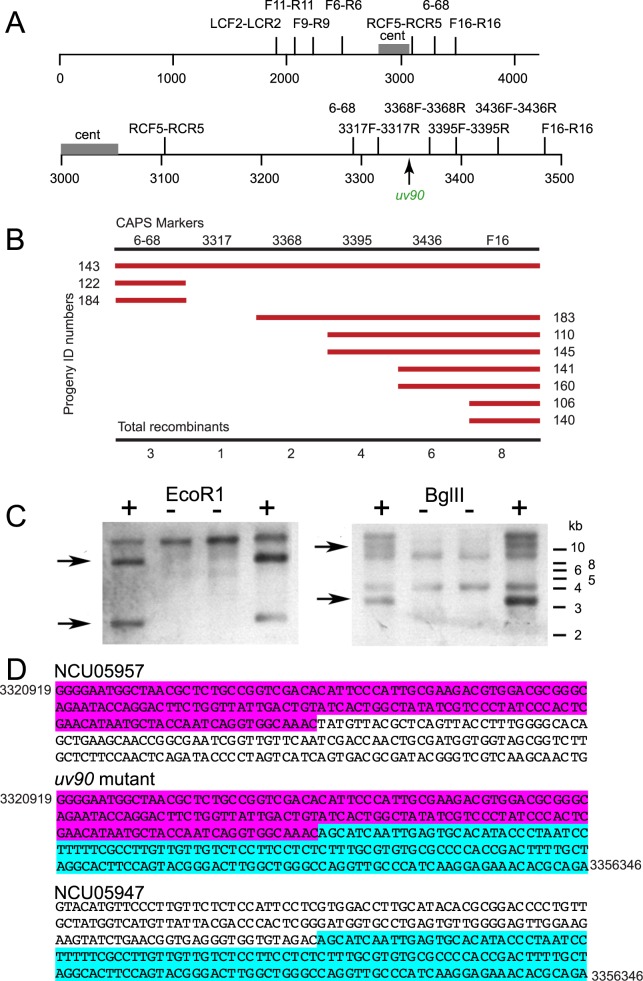
Mapping and sequencing of *uv90* mutation. (A) CAPS markers used in mapping. See S1 Table for descriptions of markers. Upper panel: Markers on linkage group (LG) VI. See S2 Table for recombination frequencies between *uv90* and these markers. Lower panel: Mapping using additional CAPS markers in expanded region of LG VI. The numbers below the lines indicate distances in kb on LG VI. Centromere indicated by “cent”. (B) Fine mapping of *uv90*. CAPS markers between 6–68 and F16 are shown on the top row. Numbers on the left and right are ID numbers of recombinant progeny. Red lines denote the markers with which the progeny were recombinant. Total numbers of recombinants are listed at the bottom. (C) Representative Southern blot comparing *uv90* wild type and *uv90* mutant. DNA from four strains, two *uv90* wild type (+) and two *uv90* mutants (-), was cut with either EcoRI (left panel) or BglII (right panel) and the blot was hybridized with a DNA probe covering the NCU05950 locus plus upstream and downstream regions. Seven additional cross progeny with the *uv90* phenotype were analyzed with similar results. Predicted bands present in *uv90* wild type and missing in the *uv90* mutant are indicated by arrows. The positions of molecular weight markers are indicated on the right. (D) Partial sequences of genes upstream and downstream of the *uv90* mutation. Highlighting indicates sequences bordering the deletion in the *uv90* mutant. Sequence coordinates are shown for the 5’ segment of NCU05957 (3320919) and 3’ of NCU05947 (3356346). The fusion point is at 3321068/3356197.

To identify the gene affected by the *uv90* mutation, we mapped the phenotype using the method of cleaved amplified polymorphic sequences (CAPS) [34]. We first crossed our lab-generated *uv90* mutant strain in the Oak Ridge background to a wild-type Mauriceville strain that carries many DNA polymorphisms relative to Oak Ridge. Using PCR-based markers designed to detect these polymorphisms in the progeny of the cross, we mapped the *uv90* mutation to a region on linkage group (chromosome) VI (Fig 1A, S1 Table, S2 Table). We then carried out fine mapping with additional CAPS markers using only progeny that had a crossover event in that region (Fig 1B). This defined the location of *uv90* as a 75 kb region around marker 3317. To identify the putative *uv90* gene, we tested the growth phenotypes of a number of strains carrying deletions of genes in this region (see S3 Table). Out of 21 ORFs identified in this region in the *N*. *crassa* genome, 16 were available as deletions from the Fungal Genetics Stock Center and the *Neurospora* Knockout Project, and one (NCU05950) showed a growth phenotype similar to the *uv90* mutant.

To confirm the presence of a mutation in NCU05950 in our *uv90* mutant, we attempted to sequence this gene in the mutant strain. Our initial attempts to obtain a PCR product from this region were unsuccessful in the mutant. Southern blots using a probe for the wild-type NCU05950 gene showed a loss of DNA fragments in this region in the mutant, suggesting a major chromosomal event such as a deletion (Fig 1C). We carried out additional PCR in this region, walking in both directions from NCU05950 in approximately 1 kb increments. PCR failed to give products until we reached genes NCU05957 upstream and NCU05947 downstream (S1 Fig, S2 Fig). Long-range PCR across this region produced products shorter than predicted in the mutant but none in the wild type, likely due to the extreme length of the predicted products (S1 Fig). We sequenced a long-range PCR product from the mutant and found a deletion of 35,128 nt (Fig 1D). This deletion removes 11 genes, including NCU05950 and two tRNAs, and partially deletes NCU05957 and NCU05947 (Fig 1D, S2 Fig).

**Fig 2:**
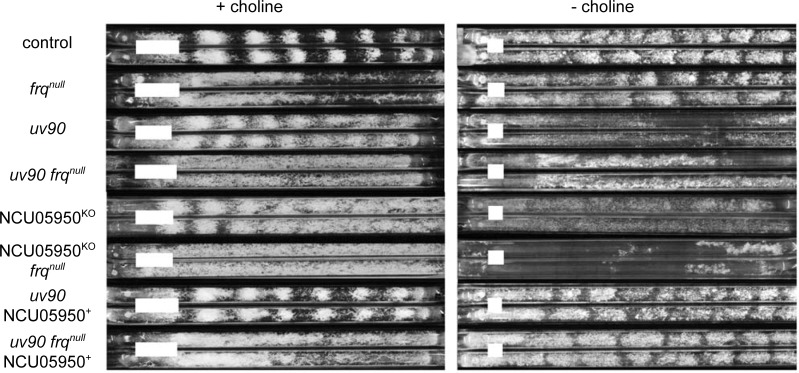
Free-running rhythmic phenotypes of NCU05950 mutants and transformants. “*frq*^*null*^” indicates a null allele of *frq*, either a point mutation *frq*^*9*^ (sets 2 and 8) or a deletion *frq*^*10*^ (sets 4 and 6). “NCU05950^KO^” indicates deletion of the NCU05950 gene. “NCU05950^+^” indicates transformant carrying a wild type copy of the NCU05950 gene at an ectopic location in the *his-3* locus. All strains, including “control”, are also *csp-1; ras*^*bd*^ *chol-1*. Strains were grown with (+, left panel) or without (-, right panel) 100 μM choline in the medium. Two representative replicate tubes for each condition are shown. Growth is from left to right. White bars indicate average growth in 24 hours. Periods and growth rates are reported in S6 Table.

**Fig 3:**
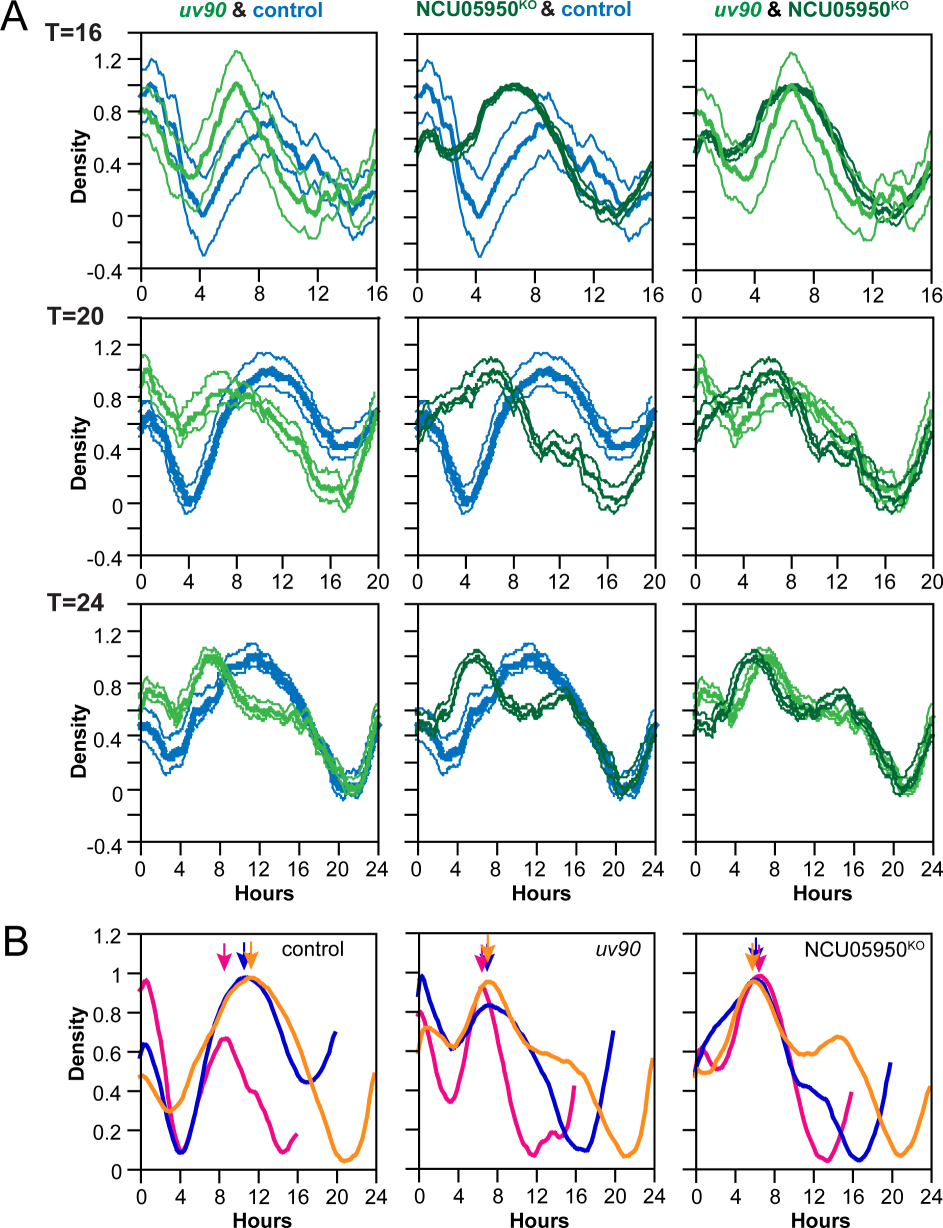
Heat pulse entrainment phenotypes of NCU05950 deletion mutant. (A) Control (pale blue), *uv90* mutant (pale green) and NCU05950^KO^ (dark green) strains were grown at 22°C and entrained to repeated 2-hour pulses of 32°C at intervals of T hours. The normalized conidiation density of an average “day” (one T-cycle) is plotted against time after the beginning of the heat pulse. T-cycles were 16 h (top row), 20 h (middle row) or 24 h (bottom row). Thin lines are ± 1 S.E.M. Six replicate tubes were averaged for each trace, either 2 or 4 cycles from each tube, so N = 12 or 24 cycles averaged. All strains, including control, are also *csp-1; ras*^*bd*^ *chol-1; frq*^*null*^. Growth media contained 100 μM choline to repair the *chol-1* defect, which is not relevant to this experimental design. (B) Comparison of different T-cycles for the heat pulse entrainment of NCU05950 deletion mutant. Data are re-plotted from (A). T-cycles were 16 h (pink), 20 h (blue) or 24 h (orange). Arrows indicate peak times.

**Fig 4:**
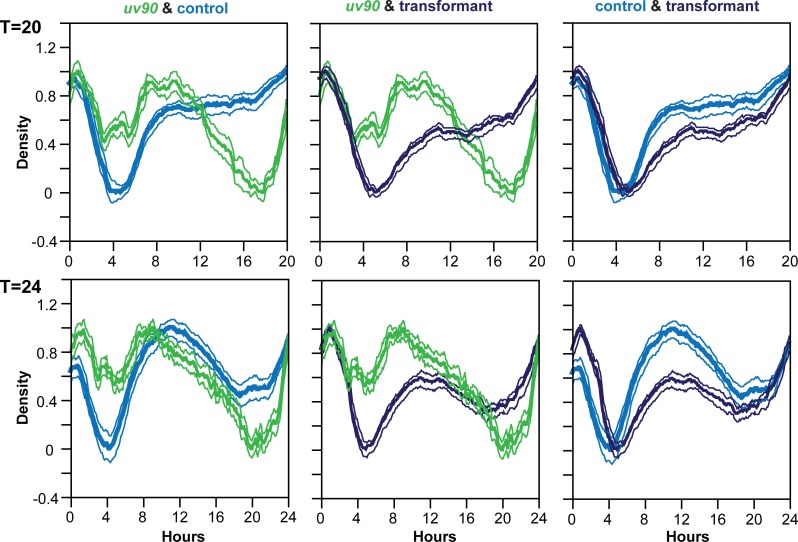
Rescue of the heat pulse entrainment phenotype of the *uv90* mutant by the NCU05950 gene. Control (pale blue), *uv90* mutant (pale green) and *uv90* mutant carrying a wild type copy of the NCU05950 gene (dark blue) were grown at 22°C and entrained to repeated 2-hour pulses of 32°C at intervals of T hours. The normalized conidiation density of an average “day” (one T-cycle) is plotted against time after the beginning of the heat pulse. T-cycles were 20 h (top row) or 24 h (bottom row). Thin lines are ± 1 S.E.M. Five replicate tubes were averaged for each trace, 2 cycles from each tube, so N = 10 cycles averaged. All strains, including control, are also *csp-1; ras*^*bd*^ *chol-1; frq*^*null*^. Growth media contained 100 μM choline to repair the *chol-1* defect, which is not relevant to this experimental design.

We confirmed that deletion of NCU05950, and only this gene, is responsible for the *uv90* phenotype by using two strategies: assaying the circadian rhythm phenotype of an NCU05950 deletion strain, and rescuing the phenotype of the *uv90* mutant by transformation with a wild-type copy of NCU05950. We crossed the NCU05950 deletion into our laboratory strains to introduce a FRQ-null mutation and the *chol-1* mutation. We assayed conidiation rhythmicity under free-running conditions and found that the phenotype of the NCU05950 deletion was very similar to the *uv90* mutant, in both the choline-supplemented and choline-deficient conditions, both with and without the FRQ-null mutation (Fig 2). The entrained rhythm of the NCU05950 deletion in a FRQ-null background was also similar to the *uv90* mutant and different from the UV90 wild-type strain (Fig 3A). (Note that the apparent troughs following the conidiation peaks do not represent entrainment, but are artifacts of the direct masking effects of heat on the development of conidiospores at the time of the heat pulse[19].) The phenotype of the deletion mutant is highlighted in Fig 3B where we have re-plotted the heat pulse profiles to compare the effects of different T-cycles within each strain. The conidiation peaks in wild-type occur at varying times after the heat pulse depending on the T-cycle, as expected from an entrained oscillator [17, 19]. In contrast, the main peaks in the *uv90* mutant and the deletion mutant occur earlier in the cycle than the wild type and at nearly the same time, demonstrating that the loss of UV90 function disables the oscillator that is revealed by the heat pulse entrainment. We then introduced a wild-type copy of NCU05950 into the original *uv90* mutant strain, both with and without the FRQ-null mutation, and found that the rhythmic phenotype was similar to the *uv90*^*+*^ wild-type, in both assays (Fig 2 & Fig 4). These results confirmed that the phenotype of the *uv90* mutant is due to a loss-of-function (null) mutation in the NCU05950 gene, and cannot be accounted for by disruption of neighboring genes.

### NCU05950 is predicted to be a component of the TOR pathway

**Fig 5:**
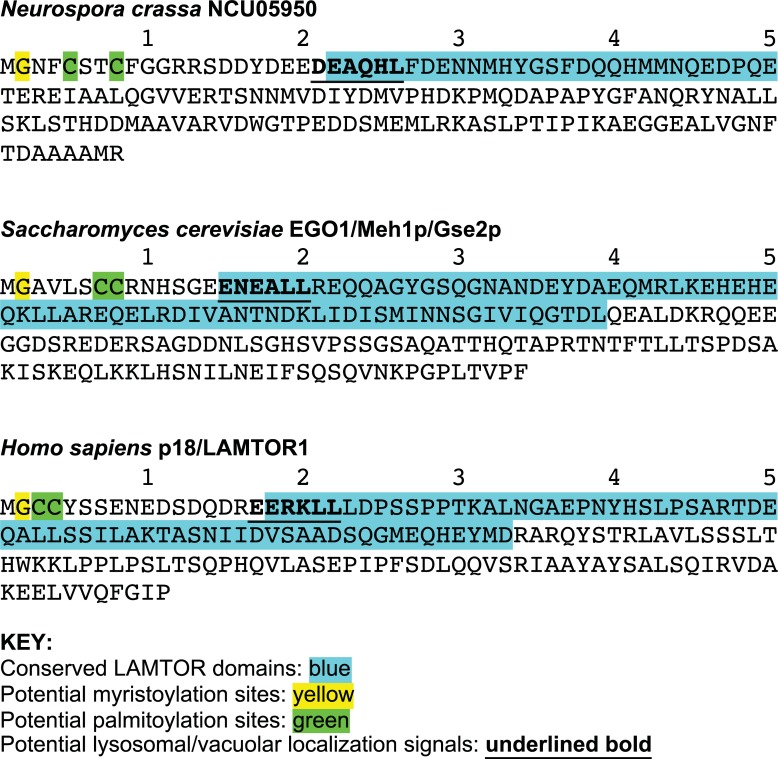
Predicted protein sequences. The NCU05950 sequence is compared to the sequences of the yeast protein EGO1/Meh1p/Gse2p (UniProt accession code Q02205) and the human protein p18/LAMTOR1 (UniProt accession code Q6IAA8). LAMTOR domains identified by NCBI Conserved Domain searches are blue; potential myristoylation sites are yellow; potential palmitoylation sites are green; potential lysosomal or vacuolar localization signals are underlined in bold.

The NCU05950 gene was not annotated in the *N*. *crassa* genome database prior to our characterization of the *uv90* mutant. Database searches using the protein sequence retrieved a number of similar proteins from other filamentous fungi; however, none of these had functional annotations (see S5 Table). We performed a search for putative protein domains and identified a conserved domain in the LAMTOR family, which includes human p18/LAMTOR1 and yeast EGO1/Meh1p/Gse2p (Fig 5 and Table 1). In yeast, EGO1 is localized to the vacuolar membrane and serves as an anchor for the EGO complex [2, 35]. Human p18/LAMTOR1 is localized to the surface of late endosomes/lysosomes to anchor the “Ragulator” complex [4]. These complexes function upstream of TORC1 and transmit nutritional signals, specifically amino acid sufficiency signals, to TORC1.

**Table 1:** Sequence comparisons of LAMTOR-family proteins.

Protein	MW (kDa)	LAMTOR domain e Value	% Alpha Helix
Human LAMTOR1	17.7	8.41e-18	37.3
Yeast EGO1	20.2	1.07e-18	39.1
*N*. *crassa* NCU05950	17.7	3.22e-05	29.1

In addition to the LAMTOR domain, the predicted amino acid sequence of NCU05950 contains elements that support the identification of the protein product as a member of the LAMTOR family (Fig 5). The predicted protein is similar in size to the human and yeast proteins (Table 1). Both human p18/LAMTOR1 and yeast EGO1/Meh1p/Gse2p are predicted to be helix-rich [36] as is NCU05950 (Table 1). Similar to LAMTOR1 and EGO1, the N-terminus of NCU05950 has two cysteines that may be sites for S-acylation (palmitoylation) and an N-terminal glycine that is a potential site for myristoylation (Fig 5). Dual acylations are known to localize some proteins to lipid rafts [37]. The human LAMTOR1 protein has been found in lipid rafts in late endosomes and these N-terminal putative acylation sites are essential for this localization [38]. The NCU05950 gene contains a sequence (DEAQHL) near the N-terminus that fits a localization signal [DE]XXXL[LI] for sorting proteins to the endosomal/lysosomal compartments in mammals, yeast, and many other organisms [39]. Both human LAMTOR1 and yeast EGO1 contain similar localization sequences (Fig 5). Pairwise protein sequence alignments identified similarities between these proteins from *N*. *crassa* and yeast, and *N*. *crassa* and human, in the N-terminal acylation sites, the lysosomal localization sequences, and the LAMTOR domains (S3 Fig).

### NCU05950 localizes to the vacuolar membrane

**Fig 6:**
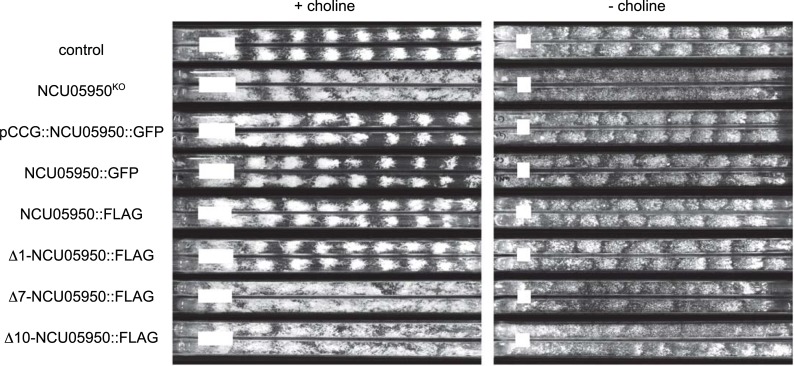
Rescue of the phenotype of the NCU05950 deletion mutant by the NCU05950-GFP and–FLAG fusion proteins. All strains, including control, have the *csp-1; ras*^*bd*^ *chol-1* genetic background. All strains except the control also carry the NCU05950 deletion allele (NCU05950^KO^). GFP- and FLAG-tagged fusion genes were inserted at the *his-3* locus of a NCU05950^KO^ strain. The pCCG fusion gene was expressed from the high-expression *ccg-1* promoter. Other fusion genes were expressed from the native NCU05950 promoter. Δ1, Δ7 and Δ10: The FLAG fusion protein of NCU05950 expressed from the native NCU05950 promoter was modified by deleting either 1, 7 or 10 amino acids after the initial methionine at the N-terminus. Strains were grown with (+, left panel) or without (-, right panel) 100 μM choline in the medium. Two representative replicate tubes for each condition are shown. Growth is from left to right. White bars indicate average growth in 24 hours. Periods and growth rates are reported in S7 Table.

**Fig 7:**
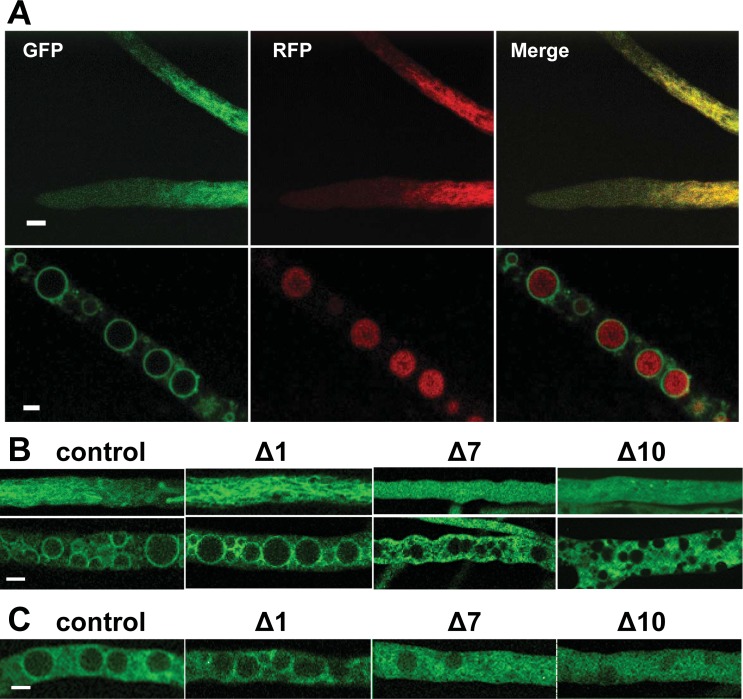
Subcellular localization of NCU05950 protein. (A) A heterokaryon expressing both a GFP fusion protein of NCU05950 and an RFP fusion protein of the vacuolar protein VAM-3 was observed in living hyphae by confocal microscopy. Top row: tips of growing hyphae. Bottom row: older hyphae containing large round vacuoles. Left column: green channel, GFP. Middle column: red channel, RFP. Right column: merged channel, red + green. Scale bars = 5 μm. (B) Subcellular localization of NCU05950 N-terminal deletion mutants. The GFP fusion protein of NCU05950 expressed from the *ccg-1* promoter was modified by deleting either 1, 7 or 10 amino acids after the initial methionine at the N-terminus (indicated as Δ1, Δ7 and Δ10). GFP localization was observed in living hyphae by confocal microscopy. Upper panels show young hyphae. Lower panels show older hyphae with mature round vacuoles. Control = full-length NCU05950. Scale bar = 5 μm. (C) Subcellular localization of GFP fusion proteins driven by the native promoter. The GFP fusion protein of NCU05950 was expressed from the NCU05950 promoter. N-terminal deletions and microscopy as for (B). Older hyphae are shown. Control = full-length NCU05950. Scale bar = 5 μm.

To determine the intracellular location of the NCU05950 protein, we constructed a GFP fusion protein expressed from the high-expression *ccg-1* promoter. This fusion protein was functional, as shown by rescue of the rhythmic phenotype in an NCU05950 deletion strain (Fig 6, row 3). As a control, we constructed a strain carrying an RFP-tagged vacuolar marker, *vam-3* (NCU06777) [40], which is a vacuolar-associated SNARE. As previously reported, [40], the RFP-VAM-3 protein was found in filamentous structures behind the tip, and inside large spherical vacuoles in older hyphal regions that were filled with large vacuoles (Fig 7A). Localization inside the vacuole may be the result of turnover of vacuolar membrane proteins and normal degradation processes [40]. The NCU05950-GFP protein co-localized with RFP-VAM-3 in hyphal tips (Fig 7A, top row). In older hyphae, NCU05950-GFP was found on the vacuolar membrane (Fig 7A, bottom row). These results localize the NCU05950 protein to the vacuole, and specifically the vacuolar membrane, in agreement with the known localization of the orthologous human and yeast proteins.

The putative acylation sites at the N-terminus of the NCU05950 protein (Fig 5) may be responsible for localizing the protein to membranes. We constructed a deletion mutant lacking the terminal glycine (amino acid #2) and we also constructed a deletion mutant lacking 7 amino acids (#2–8), which would delete both putative acylation sites. However, the 7 amino acid deletion leaves two glycines near the N-terminus, which could potentially serve as new acylation sites, so we also constructed a deletion of 10 amino acids (#2–11). GFP-fusion proteins with these deletions expressed from the *ccg-1* promoter were found to be diffusely localized in the cytosol (Fig 7B), in contrast to the vacuolar localization of the wild-type protein. Similar results were found with deletion mutants expressed from the native NCU05950 promoter (Fig 7C). S5 Fig demonstrates that the proteins with N-terminal 7- and 10-amino acid deletions were stably expressed. These results indicate that the NCU05950 gene product is a LAMTOR-like protein localized to the vacuolar membrane, probably by acyl group anchors. We also found that this vacuolar localization is required for the rhythm-related function of NCU05950: FLAG-tagged N-terminal deletion constructs expressed from the native NCU05950 promoter and missing 7 or 10 amino acids failed to rescue the defective phenotype of the NCU05950 gene deletion strain (Fig 6, rows 7 & 8). In contrast, a deletion of only the N-terminal glycine at position #2, the putative site for myristoylation, did not disrupt vacuolar localization of the protein (Fig 7B) and did not disrupt the rhythm-related function of the protein, as this deletion was able to rescue the phenotype of the gene deletion mutant (Fig 6, row 6). Similar results were found for GFP-tagged N-terminal deletion constructs expressed from the native NCU05950 promoter (S4 Fig). Quantitation of pixel brightness from confocal images (S6 Fig) confirmed that GFP-tagged proteins were concentrated at the vacuolar membranes in the control and Δ1 strains but not in the Δ7 and Δ10 strains, for both the *ccg-1* and native promoters. Acylation at the N-terminal glycine is therefore not required for correct vacuolar localization and function. (Note that the growth rate of the strain carrying Δ10-NCU05950::GFP was close to that of the strain carrying the full-length NCU05950::GFP (S8 Table) although the growth rate of the corresponding FLAG-tagged Δ10 mutant was slower than the full-length control (S7 Table).)

### NCU05950 protein levels are not rhythmic

**Fig 8:**
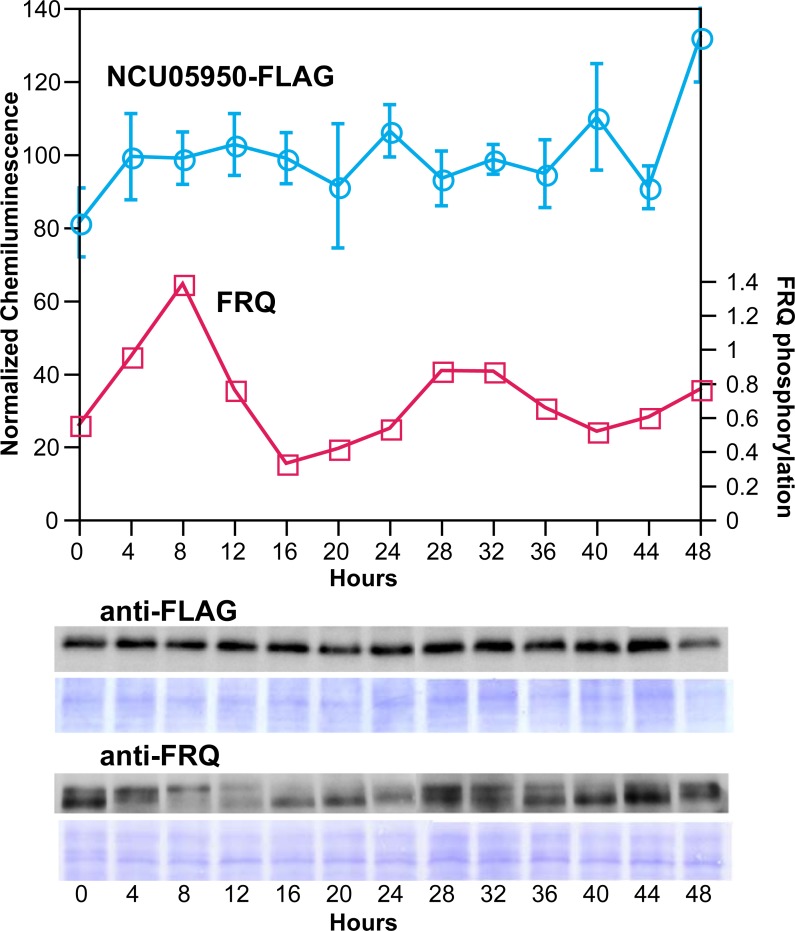
Absence of rhythmicity in NCU05950 protein levels. Samples were collected every 4 hours across two circadian cycles from cultures expressing the NCU05950-FLAG fusion protein from the native promoter. The genotype was *csp-1 his-3*^*+*^::NCU05950::FLAG; *ras*^*bd*^ *chol-1;* NCU05950^KO^. Western blotting was carried out using anti-FLAG antibody. Relative densities of chemiluminescence images within each replicate time-course were normalized to mean = 100 (left Y-axis). Data points are mean ± S.E.M. of three independent experiments. A representative blot with anti-FLAG antibody and Coomassie-stained protein is shown below. The same samples were run on a second gel and the blot is shown with anti-FRQ antibody and Coomassie-stained protein. The FRQ phosphorylation state was calculated as the ratio of the upper FRQ band (phosphorylated) to the lower FRQ band (unphosphorylated) and the ratio is plotted against the right Y-axis. Data for FRQ are from one experiment.

To determine whether the NCU05950 protein product is regulated by the circadian system, we constructed a fusion protein with a FLAG epitope tag, driven by the native NCU05950 promoter, and introduced it into the NCU05950 deletion strain. This transformant was functional and restored the wild type phenotype, as assayed by rhythmicity on race tubes (Fig 6, row 5). To assay a time-course of protein levels, cultures were grown in liquid medium in constant darkness and constant 22°C after synchronizing their clocks with a light-to-dark transition, and samples were collected at various times. The level of NCU05950 protein was assayed by immunoblotting and the protein was found at nearly constant levels across two circadian cycles (Fig 8). As a control, we assayed levels of FRQ protein in the same samples and found the expected rhythm of phosphorylation state, demonstrating that the clock in these cultures was functional (Fig 8). These experiments used samples harvested from cultures in low-glucose liquid medium in which growth is inhibited. Similar results (S7 Fig) were obtained from cultures growing rapidly on agar medium and expressing the rhythm of conidiation, using culture methods previously described [33]. We concluded that the function of NCU05950 does not require rhythmicity of the protein. We constructed GFP-tagged proteins using both the native NCU05950 promoter and the high expression *ccg-1* promoter, and we found that both types of constructs can rescue the rhythmic phenotype in the NCU05950 deletion mutant background (Fig 6, rows 3 & 4). This indicates that the native promoter is not required for the clock function of NCU05950, in agreement with our finding that rhythmicity of the protein levels is not required for clock function.

### The NCU05950 deletion mutant is defective in nutrient sensing for growth

**Fig 9:**
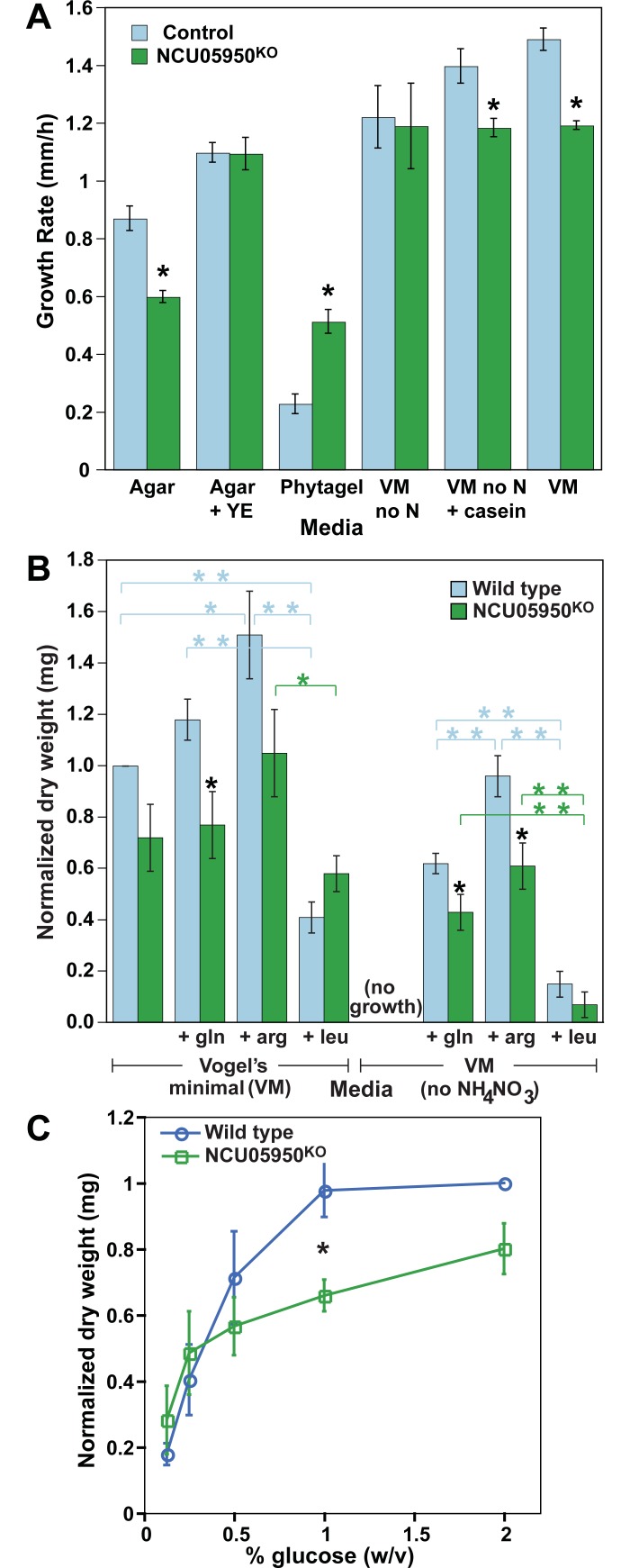
Effects of nutrients on growth rate. (A) Cultures of control (*csp-1; ras*^*bd*^) and NCU05950 deletion mutant (*csp-1; ras*^*bd*^; NCU05950^KO^) were grown on race tubes. Left three sets of columns: Media contained either agar or Phytagel as a gelling agent, and no additional nutrients except Yeast Extract (YE) in one set. Right three sets of columns: Media contained Vogel’s minimal salts plus 2% glucose and 2% agar, and either no nitrogen source (VM no N), casein hydrolysate as nitrogen source (VM no N + casein), or the usual NH_4_NO_3_ nitrogen source (VM). Values are the mean of 6–9 replicate race tubes and error bars are ± S.E.M. Stars (*) indicate statistically significant difference between control and mutant, p < 0.01. (B) Cultures of wild type or NCU05950 deletion mutant (carrying no additional mutations) were grown in liquid media with Vogel’s minimal medium (VM) and 2% glucose, with or without added NH_4_NO_3_ and added amino acids. Error bars are mean of four replicate experiments ± S.E.M. Dry weights within each experiment were normalized to the wild-type weight on VM = 1.0. Black stars (*) indicate statistically significant difference between wild type and mutant for the same growth condition, p < 0.05. Colored stars indicate statistically significant difference between growth conditions for the same strain, p < 0.05 (*) or p < 0.01 (**). (C) Effects of glucose concentration on growth rate. Cultures were grown in VM medium with NH_4_NO_3_ and various concentrations of glucose. Strains as for (B). Error bars are mean of three replicate experiments ± S.E.M. Dry weights within each experiment were normalized to the wild-type weight on 2% glucose = 1.0. Black star (*) indicates statistically significant difference between wild type and mutant, p < 0.05.

If the NCU05950 protein functions in the TOR pathway, as predicted, a null mutation might be expected to affect the growth response of the fungus to different nutritional states. We assayed the growth rate on solid agar in race tubes with various media and found differential responses of the NCU05950 wild type and the NCU05950 deletion mutant (Fig 9A). On water agar with no added nutrition, NCU05950 wild type grew faster; on phytagel with no added nutrition, the mutant was faster; when yeast extract was added to agar, they grew at the same rate. This indicates that the mutant does not have a generalized growth defect that slows growth under all conditions, but rather it responds atypically to nutritional state. Using the standard Vogel’s minimal medium (VM) with agar and glucose, both strains could grow at the same rate without added nitrogen sources (Fig 9A). The wild type increased its growth rate with added casein hydrolysate (a source of amino acids) or the standard ammonium nitrate source, while the mutant did not respond to these nitrogen sources (Fig 9A).

We repeated this analysis in a new set of strains, with the *chol-1* mutation in the background but adding sufficient choline to repair the *chol-1* defect. We included the original *uv90* mutation and two transformants carrying wild-type copies of NCU05950 at the *his-3* locus in either the NCU05950 deletion background or the *uv90* mutant background. The results (S8 Fig) indicated that in most cases the *uv90* mutant phenotype was similar to the NCU05950 deletion and the ectopic copy of the wild-type gene could reverse the growth phenotypes of the mutants. The presence of *chol-1* did not alter the phenotypes as long as there was sufficient choline supplementation.

To further refine this analysis, we grew a wild-type control and the NCU05950 deletion mutant in liquid VM medium without agar (to avoid the unknown impurities that allow growth on water agar alone) with or without ammonium nitrate, the standard nitrogen source. In mammals and yeast, nitrogen sources (typically amino acids) stimulate TORC1 activity and growth [1]. Both glutamine and leucine stimulate TOR activity in mammals and yeast [2], with glutamine being the preferred nitrogen source for yeast [41]. In *N*. *crassa*, glutamine is also the preferred amino acid nitrogen source for growth [42]. *N*. *crassa* also stores large amounts of arginine in its vacuole, and releases this nitrogen when glutamine is depleted [43]. We therefore chose to assay the effects on growth of three amino acids: glutamine (gln), arginine (arg) and leucine (leu) (Fig 9B). Both gln and arg added to VM increased the growth of the NCU05950 wild-type but had no significant effect on the growth of the mutant (Fig 9B). In media without ammonium nitrate, arg was significantly better than gln at supporting the growth of the wild-type, but there was no significant difference in the mutant (Fig 9B). Leucine inhibited the wild type in VM but had little effect on the mutant, and leucine was a poor nitrogen source for growth of both strains in the absence of ammonium nitrate (Fig 9B). Leucine and glutamine activate TORC1 through different pathways in both mammalian cells and yeast [3]. Activation of TORC1 by leucine depends on the EGO complex in yeast (Gtr1, Gtr2, Ego1 and Ego3) or the functionally homologous Ragulator-Rag complex in mammalian cells (RagA/B, RagC/D and Ragulator) while glutamine activates TORC1 by other poorly-characterized pathways [41, 44]. Our results may also indicate separate pathways for different amino acids in *N*. *crassa*.

We next tested the effects on growth rate of varying glucose concentration. The NCU05950 deletion mutant grew at the same rate as the wild type at low glucose concentrations but grew more slowly than wild type at higher concentrations of glucose (Fig 9C). Taken together, these results indicate that the NCU05950 deletion mutant is defective in its growth response to added nutrition, both nitrogen and carbon sources, and therefore the TOR pathway is implicated in growth responses in *N*. *crassa*.

## Discussion

Very little is known about the TOR pathway in *N*. *crassa* and other filamentous fungi. Rapamycin binding proteins (FKBPs) have been identified in the fungus [45, 46]. A screen of Ser/Thr kinase gene deletions in *N*. *crassa* [47] identified a homolog of yeast TOR1/TOR2 by sequence similarity, but the deletion strain is not available as a homokaryon from the Fungal Genetics Stock Center [48] suggesting that the deletion may be lethal, although this has not been rigorously tested. The same screen identified a homolog of the yeast Sch9 gene (homologous to the TOR kinase substrate S6K in mammals) and found that the deletion strain had impaired growth and impaired osmoregulation [47]. Genes encoding components of the TOR pathway have been identified throughout the fungal kingdom and are (with a few exceptions) highly conserved [49]. In the fungal plant pathogen *Fusarium graminearum*, this pathway is involved in virulence and vegetative development [50]. Our work suggests that in *N*. *crassa*, the TOR pathway is important in nutrient sensing and growth control as in other eukaryotes, and in addition plays a role in regulating circadian rhythmicity. Because the *uv90* gene is homologous in structure and function to the yeast EGO1 and mammalian LAMTOR1, and the protein is found on the vacuolar membrane, we propose naming this gene “*vta*”, vacuolar TOR-associated protein.

Connections between the TOR pathway and rhythmicity have been reported in other organisms. There are several reports that TOR pathway activity is rhythmic and most of these studies implicate the TOR pathway as an element of output pathways, communicating clock information to drive downstream rhythmic processes. In the chicken retina, phosphorylations of mTORC1 and S6K are rhythmic, and inhibition of mTORC1 dampens the amplitude of rhythmic ion channel activity [51]. In the brain of *Drosophila*, expression of the *Tor* gene shows a bimodal pattern in light/dark cycles, and rhythmic changes in neuron morphology depend on expression of *Tor* [52]. An important output of the clock is gene expression; the role of the circadian clock in regulating gene transcription is well known, and recent findings have shown that the clock is also able to regulate specific genes at the level of translation in several organisms [53, 54] including *Neurospora* [55]. The TOR pathway can play a role in this translational regulation: in mouse cells, mTORC1 activity is rhythmic [56], which drives rhythmic phosphorylation of 4E-BP1 and rhythmic translation of proteins [57]. Rhythmic activation of the mTORC1 target S6K in mice causes rhythmic phosphorylation of the canonical clock protein BMAL1 [58]; this clock protein has now been shown to be a translation factor that regulates rhythmic translation independently of its transcriptional function [58].

Evidence for a role for TOR in the clock mechanism itself is sparse. In *Drosophila*, TORC1 activity is rhythmic [59]. Overexpression of TOR or S6K lengthens the period [60], while silencing the *Tor* gene decreases the period [52]. S6K appears to interface with the clock TTFL through phosphorylation of the kinase SGG, which in turn phosphorylates and regulates TIM protein [60]. In mice, knockout of the tuberous sclerosis complex gene *Tsc2* (an inhibitor of mTORC1) results in changes in free-running period and phase-shifting behavior of circadian outputs. These effects apparently result from an increase in BMAL1 levels due to effects of *Tsc2* knockout on translation and protein turnover, since the clock effects can be reversed by decreasing BMAL1 levels [61].

Our results with the *uv90* gene provide evidence that the TOR pathway and nutritional sensing play a role in the mechanism of rhythmicity in *N*. *crassa*, both in the presence and absence of a functional FRQ/WCC TTFL. We have previously identified the clock-affecting PRD-1 gene product as an RNA helicase located in the nucleus [62]. The localization of PRD-1 is responsive to glucose in the growth medium, and the *prd-1* mutant is defective in nutritional compensation of the circadian period [63]. Mutations in both *uv90* and *prd-1* compromise rhythmicity in FRQ-less conditions [33, 64], implicating nutritional sensing and metabolism in this system. Because rhythmicity can be studied in this organism in the absence of the TTFL, *N*. *crassa* provides a model system for dissecting the role of the TOR pathway in rhythmicity and identifying the molecular mechanisms of the circadian metabolic network. The TOR pathway has been seen as a one-way signaling pathway to promote increased translation in response to extracellular signals of nutritional availability; however, there are indications that the TOR pathway also responds to internal nutritional status and translational rate in a negative feedback loop [5]. This suggests the intriguing possibility that this feedback loop might provide the substrate for the evolution of the oscillator that functions in the absence of the canonical TTFL.

## Materials and methods

### Strains

The Oak Ridge (OR) and Mauriceville (MV) wild types used for mapping were FGSC 4200 and FGSC 2225 and were obtained from the Fungal Genetics Stock Center (FGSC, Kansas State University, Manhattan, KS)[48]. Strains carrying gene deletions produced by the *Neurospora* Functional Genomics Project [65] were also obtained from FGSC. Other strains were generated in our laboratory by standard crossing techniques as previously described [20]. The *uv90* mutation was identified in a mutagenesis screen using UV light as previously described [33]. Strains carrying the *uv90* mutation were identified by phenotype (damped or arrhythmic conidiation, depending on genetic background), and by molecular genotype (failure to produce PCR products from NCU05950-specific primers). Genotypes of multiple mutant strains were identified by various criteria as previously described [62]. The *ras*^*bd*^ mutation renders conidiation resistant to inhibition by high CO_2_ levels and allows assay of rhythmicity in closed growth tubes. The *chol-1* mutation impairs synthesis of the lipid phosphatidylcholine and is carried in most of our strains to allow assay of the FRQ-less rhythm in choline-depleted cultures. When *chol-1* is supplemented with choline, the *chol-1* defect is repaired and the cultures are indistinguishable from *chol*^*+*^ strains in our assay system. This allows us to assay FRQ-less rhythms that do not depend on choline depletion, such as heat pulse entrainment behavior, in the same genotypes.

It should be noted that the *csp-1* mutation is included in the genotype of most of the strains used in this work. The *csp-1* mutation was identified [66] as a morphological mutation that prevents the separation of conidiospores from aerial hypha, hence the name “conidial separation” (csp). This mutation was introduced a number of years ago into strains utilized for circadian rhythm work [67] as it greatly reduces the contamination of cultures with free-flying spores that can occur with *csp-1* wild-type genotypes. The CSP-1 gene product has recently been identified as a transcriptional repressor that affects transcription of a number of genes in response to light [68]. (Note that our experiments are all conducted in constant darkness, removing the complications of light regulation.) CSP-1 also dampens the amplitude and shifts the phase of a number of rhythmically-transcribed genes [69]. The gene NCU05950 that we have characterized in this paper is reportedly not controlled by *csp-1* [68]. The *csp-1* mutation we have used inactivates the DNA binding domain of the CSP-1 protein and is likely to be a null mutation [70, 71]. This mutation causes a small, 1 h decrease in the period of the circadian conidiation rhythm (relative to wild type) on maltose media [25]. A true deletion mutant of *csp-1* decreases the period by about 2 hours (relative to wild type) on high glucose media but not on low glucose media [71]. This deletion similarly decreases the period of the rhythm of a *frq-luc* reporter gene by about 2 h but does not dampen the amplitude [71], indicating that the CSP-1 gene product does not play a major role in the FRQ/WCC TTFL but does provide a small amount of feedback on nutritional state. The CSP-1 gene product therefore appears to primarily function in transcriptional control of output pathways. Our initial report of the phenotype of the *uv90* mutation [33] demonstrated that the *uv90* mutation dampens the amplitude of the circadian oscillator, as shown by increased responses to light and temperature phase-resetting and dampening of the rhythm of FRQ protein [33], and therefore the *uv90* mutation is not acting on output pathways but rather on the central clock mechanism. The work reported in this paper was initiated before the molecular characterization of *csp-1*, and this lab has continued to use the genetic background in which the *uv90* mutation was generated and characterized in order to maintain consistency between experiments. In every case in which the phenotype of *uv90* or the NCU05950 knockout is assayed, the control strains carry the same genetic background, including *csp-1* where indicated, allowing us to conclude that the effects we report are due to the NCU05950 gene and not a variable genetic background. The growth experiments in liquid media in Fig 9B & 9C used *csp-1*^*+*^ strains so that uniform conidial suspensions could be used for inoculation of liquid cultures, and the results using VM medium in race tubes with *csp-1* (Fig 9A) and in liquid VM using *csp-1*^*+*^ (Fig 9B) are comparable.

The phenotypes of the *uv90* mutation and the NCU05950 knockout have not been assayed in genetic backgrounds other than those indicated in this paper, and therefore we do not have evidence one way or the other as to whether the phenotypes are dependent on a particular genetic background. For the purposes of this paper, the rhythm phenotypes have been used to map the position of the *uv90* mutation and to demonstrate the repair of the mutant phenotype by a wild-type copy of the NCU05950 gene, confirming its identity. Therefore the question of whether or not the phenotypes are different in different genetic backgrounds is not relevant to the central findings reported in this paper.

### Assays for rhythmicity and growth rate

For assaying rhythmicity of conidiation, cultures were grown on solid agar medium in glass growth tubes (“race tubes”) on media containing 1x Vogel’s salts, 0.5% maltose, 0.01% arginine and 2% agar, as previously described [62]. Entrainment to heat pulses and data analysis were carried out as previously described [19, 62, 64].

For assaying growth rates on various media in race tubes (Fig 9A), growth rates were calculated for each day of growth and it was found that growth rates increased until cultures reached maximum or steady growth rates at day 4 (or 6 for Phytagel which grew more slowly). Therefore growth rates are reported for day 4 only, or day 6 for Phytagel. For S8 Fig, uneven growth patterns were observed for some media. Several days were averaged for some sets, as follows: agar, days 2,3,4 & 5; VM no N, days 2–3; VM no N + casein, day 1 only; and VM, days 4–5. Means were compared using the unpaired two-tailed Student’s T-test.

For assaying growth rates in liquid media, cultures were grown in 1 ml of appropriate media in 24 well microtiter plates. Strains used were the OR wild type or the NCU05950 deletion mutant with no additional mutations. For inoculation, spore suspensions were made in water and counted so that 500 spores in 0.1 ml were inoculated into 1 ml medium in each well. Cultures were grown at 25°C in a 12:12 LD (light/dark) cycle. Preliminary time-course experiments showed that the cultures were growing linearly at 48 hours so this was chosen as the time point at which the cultures were harvested. The mycelia were harvested over nylon net filters, washed with water, and dried for 48 hours at 80°C in microfuge tubes that had been pre-dried and weighed empty. The dried samples were weighed and the empty tube weights subtracted to give the net dry weights. Three replicates of each condition were averaged to give one value for each independent experiment, and means were calculated from three or four independent experiments. Means were compared using the unpaired two-tailed Student’s T-test.

### Mapping the *uv90* mutation

A cross was made between the original *uv90* strain generated by mutagenesis, genotype *csp-1; chol-1 ras*^*bd*^; *uv90*; *frq*^*10*^ (*mat a*) in the Oak Ridge (OR) background, and a *ras*^*bd*^ (*mat A*) strain, as previously reported [33]. From the progeny of this cross, a *csp-1; ras*^*bd*^; *uv90* (*mat a*) strain was chosen as the parent in a further mapping cross to the Mauriceville (*mat A*) (MV) wild-type. Progeny from the second cross were phenotyped for *csp-1* by failure of spore separation, and *csp-1* progeny were inoculated onto race tubes to assay growth rate and conidial banding to determine *ras*^*bd*^ and *uv90* phenotypes.

Single nucleotide polymorphisms (SNPs) between the OR and MV strains were used to map the location of the *uv90* gene. The cleaved amplified polymorphic sequence (CAPS) marker method [34] uses specific SNPs that either remove or create an enzyme restriction site in either the OR or MV strain. PCR products are amplified from genomic DNA of progeny from a cross between the mutation of interest in the OR background, and the wild-type MV strain. PCR products are cleaved with the restriction enzyme of interest and run on gels; cleaved or uncleaved PCR products indicate which parent contributed that region of the genome, and the recombination frequency between that CAPS marker and the mutation of interest can be determined.

Marker 6-68-MspI was from Jin et al. [34]. Marker F9-R9 was from Lambreghts et al [70]. Marker F6-R6 was developed using the method of Jin et al [34] modified as previously described [62]. Other markers were developed using locations of SNPs identified by Pomraning, Smith and Freitag, and posted on the website of the Fungal Genetics Stock Center [72]. CAPS markers were designed to be approximately 400–500 base pairs in length with single restriction sites for the enzyme affected by the SNP. Primers were designed to have GC contents of 50–55%, melting temperatures (Tm) of 58–65 ^o^C, and lengths of 20–22 nucleotides.

### Southern analysis

Southern analysis was carried out according to standard procedures [73]. For Fig 1C, genomic DNA was isolated from two *uv90*^*+*^ wild type strains of genotypes *csp-1; chol-1 ras*^*bd*^ (*mat a*) and *csp-1; chol-1 ras*^*bd*^; *frq*^*10*^ (*mat a*), and two *uv90* mutant strains of genotypes *csp-1; chol-1 ras*^*bd*^; *uv90* (*mat a*) and *csp-1; chol-1 ras*^*bd*^; *uv90*; *frq*^*10*^ (*mat a*). Seven additional strains with the *uv90* phenotype were also analyzed. DNA was digested with the appropriate restriction enzyme, separated by electrophoresis on 0.8% agarose gels, denatured and transferred to nylon membranes. The probe (2883bp) consisted of the NCU05950 gene plus 1kb upstream and downstream sequence, amplified from pBM61-NCU05950 plasmid DNA using the following primers: TCCGGGCCCTCATCTTCCCTGTTGAACTCGTG and ATAAGAATGCGGCCGCCGAATGTTGCATGTCCTTCCA. The labeled probe was made using a digoxigenin labeling kit (DIG High Prime DNA Labeling and Detection Starter Kit II, Roche) according to the manufacturer’s instructions and was hybridized to the membrane. After washing the membrane, the signal was detected using an immunochemical method and chemiluminescence was recorded on film or by a CCD camera.

### Cloning the NCU05950 gene and rescue of the *uv90* mutant phenotype

The genomic sequence of NCU05950 along with 1kb upstream and downstream (2883bp in total) was amplified from genomic DNA of a *csp-1; chol-1 ras*^*bd*^; *frq*^*10*^ (*mat a*) strain using the same primers as for Southern analysis. The PCR product was inserted into the ApaI-NotI site of the *his-3* targeting plasmid pBM61 [74]. The presence of the NCU05950 gene on the recombinant plasmid (named pBM61-NCU05950) was confirmed by PCR, restriction digestion and sequencing. Conidiospores of a *csp-1 his-3; chol-1 ras*^*bd*^; NCU05950^KO^ (*mat a*) strain (carrying the deletion of NCU05950) were transformed with the plasmid by electroporation and *his-3*^*+*^ transformants were selected. Successful integration at the *his-3* locus was confirmed by PCR using combinations of plasmid-specific and gene-specific primers. Heterokaryotic transformants were purified by making uninucleate microconidia as previously described [62]. Homokaryotic transformants, genotype *csp-1 his-3*^*+*^::NCU05950; *chol-1 ras*^*bd*^; NCU05950^KO^ (*mat a*), were confirmed by using PCR primers specific for the *his-3* locus.

Positive identification of NCU05950 as *uv90* required demonstrating that the NCU05950 wild type gene could rescue the defective phenotype of the *uv90* mutant. The simplest method would be to transform a *his-3; uv90* strain with the *his-3* targeting pBM61-NCU05950 plasmid, as was done with the *his-3;* NCU05950^KO^ strain. For unknown reasons, we were unable to construct a *his-3; uv90* double mutant strain in spite of a number of attempts with various combinations of parental strains. Therefore, we adopted a different strategy. A strain of genotype *chol-1 ras*^*bd*^; *uv90*; *frq*^*9*^ (*mat A*) was constructed to act as female parent in a cross to a *csp-1 his-3*^*+*^::NCU05950; *chol-1 ras*^*bd*^; NCU05950^KO^ (*mat a*) transformant in order to introduce the NCU05950 sequence into the *uv90* mutant. The *csp-1* mutation, which is carried in most of our laboratory strains, was omitted from the female parental strain because *csp-1* makes a poor female parent. The null *frq*^*9*^ allele was introduced instead of the *frq*^*10*^ deletion to allow selection against the NCU05950^KO^ allele by hygromycin sensitivity; both the *frq*^*10*^ and NCU05950^KO^ alleles confer hygromycin resistance and this would complicate the analysis of progeny. All progeny from the cross carried *chol-1* and *ras*^*bd*^ (present in both parents). Progeny were screened for the *csp-1* phenotype and for hygromycin sensitivity. All *csp-1* and *uv90* mutant (hygromycin sensitive) progeny were tested for rhythm phenotypes on race tubes with or without 100μM choline and the four expected classes of progeny were obtained: with or w/o the *frq*^*9*^ mutant allele (identified by absence or presence of rhythmicity with choline) and with or w/o *his-3*^*+*^::NCU05950 (identified by presence or absence of rhythmicity without choline).

### Construction of GFP and RFP fusion strains and N-terminal deletion mutants

To construct a highly-expressed fusion of NCU05950 and GFP, the NCU05950 coding sequence (672bp) was amplified by PCR from the previously constructed pBM61-NCU05950 plasmid using the following primer pair: GCTCTAGAATGGGCAACTTTTGCTCAACC and CCTTAATTAAACAGCGCATGGCGGCAGCG and inserted into the XbaI-PacI site of the *his-3* targeting pCCG::C-Gly::GFP plasmid [75] between the highly-expressed *ccg-1* promoter and the 10X Gly linker upstream of the GFP sequence. To construct a fusion protein expressed from the native NCU05950 promoter, the coding sequence with upstream region (1651bp total) was amplified from pBM61-NCU05950 using the following primers: ATAAGAATGCGGCCGCTCGTGGTCCGTTCCTGATG and CCTTAATTAAACAGCGCATGGCGGCAGCG and inserted into the NotI-PacI site of the *his-3* targeting pCCG::C-Gly-GFP plasmid, removing the *pccg-1* sequence and inserting the NCU05950 sequence upstream of the 10X Gly linker and GFP sequence. Plasmids were transformed by electroporation into conidiospores of a *csp-1 his-3; chol-1 ras*^*bd*^; NCU05950^KO^ (*mat a*) strain. Heterokaryotic transformants were purified by microconidia preparation and the presence of the cloned gene in the homokaryons was confirmed by PCR. Expression of the fusion proteins was confirmed by Western blotting using anti-GFP antibodies. Both the overexpressing and native promoter strains produced a band corresponding to 44.6kDa, the predicted size of the NCU05950-GFP fusion protein.

The RFP-VAM-3 strain was constructed by transforming the pRFP-Vam-3 plasmid [40] into a *csp-1 his-3; chol-1 ras*^*bd*^ (*mat a*) strain to construct a strain of genotype *csp-1 his-3*^+^::pCCG-1::tdimer2(12)::*vam-3*^*+*^; *chol-1 ras*^*bd*^ (*mat a*) that expresses the RFP::VAM-3 fusion protein from the high expression *ccg-1* promoter.

Strains carrying deletions of 1 (G), 7 (GNFCSTC) or 10 (GNFCSTCFGG) amino acids of NCU05950-GFP expressed from the *ccg-1* promoter were constructed by replacing either the first 2, first 8 or first 11 amino acid codons (see Fig 5) with an initiator methionine codon. The pCCG::NCU05950::C-Gly::GFP plasmid was used as the template for PCR, and XbaI and PacI restriction sites were introduced at either end. The following forward primers were used: for 1-deletion, GCTCTAGAATGAACTTTTGCTCAACCTGCTTCGGC; for 7-deletion, GCTCTAGAATGTTCGGCGGTAGGAGGAGCGATGAC; for 10-deletion, GCTCTAGAATGAGGAGGAGCGATGACTACGATGAG. The reverse primer for all three constructs was CCTTAATTAAACAGCGCATGGCGGCAGCG GCATCGG. PCR products were inserted into the XbaI-PacI site of the pCCG::C-Gly::GFP plasmid.

Strains carrying deletions of 1, 7 or 10 amino acids of NCU05950-GFP expressed from the native promoter were constructed by utilizing a SanDI restriction site (GGGACCC) 261–267 nt upstream of the start codon, and a BglII restriction site (AGATCT) 328–333 nt downstream. Gene sequences between these two sites, but missing either 1, 7 or 10 amino acid codons as described above, were commercially synthesized (Integrated DNA Technologies, Inc., Coralville, IA, USA) and inserted between the SanDI and BglII sites on the GFP plasmid described above harbouring NCU05950-GFP expressed from its native promoter. Plasmids were transformed by electroporation into conidiospores of a *csp-1 his-3; chol-1 ras*^*bd*^; NCU05950^KO^ (*mat a*) strain. Heterokaryotic transformants were purified by microconidia preparation and the presence of the cloned gene in the homokaryons was confirmed by PCR analysis.

### Fluorescence microscopy

Expression of fluorescence from GFP- and RFP-tagged strains was observed in live-cell cultures by confocal microscopy. For Fig 7A, heterokaryons between the RFP-VAM-3 and NCU05950-GFP strains were made by co-inoculating both strains into one culture tube. Several heterokaryon cultures were screened by confocal microscopy to choose one with approximately equal fluorescence intensity in the green and red channels for observation of co-localization. Cultures were grown on agar-covered microscope slides and living hyphae were observed by confocal microscopy as previously described [76]. Young hyphae were imaged using a Bio-Rad MRC 600 confocal laser scanning microscope with a 60x oil-immersion objective. Image contrast was adjusted in Photoshop. Images of older hyphae were collected on a Leica DMI 3000 inverted microscope equipped with an oil immersion objective (Leica, 100×, NA 1.40) with a piezo objective drive from Physik Instruments and attached to an Andor DSD2 confocal scanner. System was integrated and supplied by Quorum Technologies (Guelph, Ontario). Images were processed with Volocity software.

For Fig 7B, strains with deletions of 1, 7 or 10 amino acids of the NCU05950 sequence were used. The strains were otherwise similar to the GFP fusion strain in Fig 7A. For Fig 7C, deletion strains were similar to 7B but the GFP fusion genes were expressed from the native NCU05950 promoter. Living hyphae were observed using a Zeiss LSM 700 confocal microscope under 40x oil immersion. Image contrast was adjusted in Photoshop.

For S6 Fig, pixel brightness was quantitated to demonstrate localization of the NCU05950-GFP fusion protein at the vacuolar membrane. Three images for each strain were analyzed, and either one, two or three vacuoles per image were quantitated, for a total of six vacuoles per strain. For each vacuole, transects were constructed by drawing six lines of 5 pixels wide starting from the interior of the vacuole and extending across the membrane into the cytosol. Transects were approximately the length of the vacuole diameter and were distributed around the vacuolar circumference, avoiding nearby organelles in the cytosol. The Plot Profile function of ImageJ (NIH) was used to measure the mean gray value of the pixels and generate a profile of the pixel brightness of each transect. The profiles of the six transects for each vacuole were averaged. Each mean profile was normalized by dividing each pixel value by the average of the entire profile. The six normalized mean profiles from six vacuoles per strain were then averaged. Because vacuoles were different sizes and the transects were different lengths, it was necessary to align the six transects so the vacuolar membranes were coincident. This was done by aligning the normalized profiles so that the values closest to the average of 1.0 on the upward slope of the profile (corresponding to the interior edge of the vacuolar membrane) were aligned. The averages of the six normalized transects were then plotted for S6 Fig with the SEM calculated for N = 6.

### Construction of FLAG-tagged NCU05950 protein and immunoblotting

For quantitation of NCU05950 protein levels by immunoblotting, a FLAG epitope tagged fusion protein expressed from the native promoter was constructed using the same primers and strategy as the GFP fusion protein, but the PCR product was inserted into the *his-3* targeting pCCG::C-Gly-3XFLAG plasmid [75]. The plasmid was transformed into a *csp-1 his-3; chol-1 ras*^*bd*^; NCU05950^KO^ (*mat a*) strain by electroporation and transformants were purified by preparation of microconidia.

Strains carrying deletions of 1, 7 or 10 amino acids of NCU05950-FLAG expressed from the native promoter were constructed by utilizing a SanDI restriction site 261–267 nt upstream of the start codon, and a BglII restriction site 328–333 nt downstream. Gene sequences between these two sites, but missing either 1, 7 or 10 amino acid codons as described for the GFP fusion strains, were commercially synthesized (Integrated DNA Technologies, Inc., Coralville, IA, USA) and inserted between the SanDI and BglII sites on the FLAG plasmid described above.

Samples for immunoblotting were grown either in conditions of slow growth in low-glucose liquid media or growing rapidly on top of cellophane overlaid on solid agar. Liquid media cultures were grown using methods modified from Edgar et al. [31]. Conidiospores of the transformant were inoculated into 1 ml high-glucose liquid medium (VM plus 2% glucose, 0.5% arginine, 10 ng/ml biotin, 0.2% Tween 80, 100 μm choline) in wells of 24-well microtiter plates and allowed to grow at 25°C in constant light (LL) for 2 days. The resulting hyphal mats were transferred to 50 ml of low-glucose medium (VM plus 0.03% glucose, 0.05% arginine, 10 ng/ml biotin, 100 μm choline) in 100 ml flasks and shaken at 150 rpm on an orbital shaker. Cultures were transferred to constant dark (DD) at various times to set the circadian clock at different phases, from 0 to 48 hours in DD. Samples were harvested by vacuum filtration after 48–56 hours of growth in flasks, frozen in liquid N_2_ and stored at -80°C.

Cultures on sold agar were grown and harvested as described previously [33]. Cultures were grown in 150 mm Petri plates at 22°C on top of cellophane overlaid on the same medium used in race tubes, containing 1x Vogel’s salts, 0.5% maltose, 0.01% arginine and 2% agar. Cultures were initiated in constant light, transferred to DD at various times, and harvested after 72 hours of total growth. The times of transfer to DD were varied so that the time in darkness at harvest varied from 0 to 48 h. Cultures were harvested by scraping off the final 1 cm of growth at the colony edge, and samples were frozen in liquid N_2_.

For electrophoresis, samples were ground in liquid N_2_ and total protein was extracted in boiling SDS buffer (50 mM tris pH 6.8, 2% SDS, 10% glycerol, 5 mM EDTA, 1 mM PMSF). Protein concentration was assayed by Bio-Rad DC Protein Assay and 0.1 M DTT and 0.1% bromphenol blue were added before electrophoresis. 20 μg protein was run on 15% acrylamide gels in Tris-glycine-SDS buffers, blotted to Immobilon-P PVDF membranes, and immunodetected using a monoclonal anti-FLAG M2 antibody (Sigma-Aldrich) and HRP-conjugated goat anti-mouse secondary antibody (Origene). For quantitation of FRQ protein, 50 μg protein samples prepared as above were run on a 7.5% gel, blotted to Immobilon-P and detected using anti-FRQ monoclonal primary antibody and HRP-conjugated goat anti-mouse secondary antibody. The FRQ antibody was generously supplied by M. Merrow [77] and M. Brunner. Chemiluminescence was detected with a CCD camera and quantitated with ImageJ software. UV90 protein was normalized against total protein by staining the membrane with Coomassie Blue after immunodetection. FRQ phosphorylation state was quantitated by calculating the ratio between the density of the upper band of FRQ protein (highly phosphorylated) to the density of the lower FRQ band (relatively dephosphorylated).

### Bioinformatics

*N*. *crassa* sequences were retrieved from the FungiDB database (http://fungidb.org/fungidb/) [78]. Database searches for homologs were carried out using NCBI BLAST software (https://blast.ncbi.nlm.nih.gov/Blast.cgi) [79]. Protein domains were identified using the NCBI Conserved Domain search tool (https://www.ncbi.nlm.nih.gov/Structure/cdd/cdd.shtml) [80]. Pairwise alignments of protein sequences were made using EMBOSS Needle software (http://www.ebi.ac.uk/Tools/psa/) [81]. Protein secondary structure predictions were made using SPIDER2 software (http://sparks-lab.org/server/SPIDER2/) [82].

## Supporting information

S1 Table CAPS markers used in mapping the *uv90* mutation. (PDF)

S2 Table Recombination frequencies between the *uv90* mutation and CAPS markers. (PDF)

S3 Table ORF candidates for the *uv90* gene located on LG VI, from 3.293 to 3.369 Mbp. (PDF)

S4 Table PCR primers used to analyze the UV90 mutation. (PDF)

S5 Table Top ten sequences similar to NCU05950. (PDF)

S6 Table Periods and growth rates of NCU05950 mutants and transformants. (PDF)

S7 Table Periods and growth rates of NCU05950-GFP and–FLAG fusion proteins. (PDF)

S8 Table Periods and growth rates of GFP-tagged N-terminal deletion strains. (PDF)

S1 Fig PCR of *uv90* mutant allele. Representative gels of PCR products derived from the regions around the *uv90* deletion. Top row: Primer pairs on either side of the deletion breakpoints showing absence or presence of products in the *uv90* mutant. Bottom row: long-range PCR across the breakpoint showing presence of shorter products in the *uv90* mutant. Two strains of each genotype (*uv90* wild-type “control” and *uv90* mutant) are shown. Genotypes are: control 1, *csp-1; chol-1 ras*^*bd*^; control 2, *csp-1; chol-1 ras*^*bd*^; *frq*^*10*^; *uv90* mutant 1, *csp-1; chol-1 ras*^*bd*^; *uv90*; *uv90* mutant 2, *csp-1; chol-1 ras*^*bd*^; *uv90*; *frq*^*10*^. PCR primers are listed in S4 Table and are mapped in S2 Fig.
(PDF)

S2 Fig Map of PCR reactions across chromosome VI in the *uv90* region. Nucleotide positions on chromosome VI and gene sizes are shown for genes. Marks are at 1 kb intervals. PCR primers shown above the chromosome are listed in S4 Table.
(PDF)

S3 Fig Sequence alignments of LAMTOR-related proteins. (PDF)

S4 Fig Rescue of the phenotype of the NCU05950 deletion mutant by GFP-tagged N-terminal deletion strains. All strains, including control, have the *csp-1; ras*^*bd*^ *chol-1* genetic background. All strains except the control also carry the NCU05950 deletion allele (NCU05950^KO^). GFP-tagged fusion genes were inserted at the *his-3* locus of a NCU05950^KO^ strain and were expressed from the native NCU05950 promoter. Δ1, Δ7 and Δ10: The GFP fusion protein of NCU05950 was modified by deleting either 1, 7 or 10 amino acids after the initial methionine at the N-terminus. Strains were grown with (+, left panel) or without (-, right panel) 100 μM choline in the medium. Two representative replicate tubes for each condition are shown. Growth is from left to right. White bars indicate average growth in 24 hours. Periods and growth rates are reported in S8 Table.
(PDF)

S5 Fig Expression controls for GFP-tagged N-terminal deletion constructs. Top panel: Western blot of GFP-tagged proteins expressed from the *ccg-1* promoter. The expected size of the tagged full-length protein is 44.6 kDa. Strains are described in Fig 7B. Lane A: molecular weight markers. Lane B: full-length GFP-tagged protein. Lane C: Δ7 deletion. Lane D: Δ10 deletion. Lane E: untagged control strain. Bottom panel: Confocal microscopy of GFP-tagged protein expressed from the native promoter. Strains and conditions as for Fig 7C. Top row: N-terminal Δ10 deletion. Bottom row: untagged control strain, showing autofluorescence. Left column: Images taken with the same microscope brightness and contrast settings. Right column: Images with levels adjusted in Photoshop to show autofluorescence of control strain.
(PDF)

S6 Fig Localization of NCU05950-GFP near vacuolar membranes. Images related to Fig 7 were analyzed by quantitating pixel brightness across transects drawn from the interior of vacuoles across the vacuolar membrane into the cytosol. Pixel number zero is in the interior of the vacuole. Scale: 10 pixels = 4 μm. Six vacuoles per strain were quantitated. Error bars are mean ± SEM (N = 6). Left column: NCU05950-GFP driven by the *ccg-1* promoter. Right column: NCU05950-GFP driven by the native promoter. Top row: full-length NCU05950 gene fused to GFP (control). Rows 2, 3 and 4: N-terminal deletions of NCU05950 fused to GFP, deleting 1, 7 or 10 amino acids.
(PDF)

S7 Fig NCU05950 protein levels on agar medium. Cultures were grown on top of cellophane overlaid on solid agar medium. Samples were collected every 4 hours, processed for immunoblotting, and analysed as for Fig 8. Data points are mean ± S.E.M. of three independent experiments.
(PDF)

S8 Fig Reversal of nutrient growth effects by transformation with wild-type NCU05950 gene. Strains were grown on race tubes containing various growth media as described in Fig 9. All strains carried the *csp-1; ras*^*bd*^ *chol-1* background and were grown with 100 μM choline added to the media. *uv90* strains are the original *uv90* mutant. NCU05950^ko^ strains are the deletion mutant. Strains marked T (for transformant) also carried a wild-type copy of NCU05950 inserted at the *his-3* locus. Values are the mean of three replicate race tubes and error bars are ± S.E.M. Stars (*) indicate statistically significant difference between mutant and control, or between transformant and corresponding mutant; *, p < 0.05; **, p < 0.01; n.s., not significantly different.
(PDF)
