# Supplementary material for: A component of the TOR (Target Of Rapamycin) nutrient-sensing pathway plays a role in circadian rhythmicity in Neurospora crassa
Source: PLoS Genet. 2018 Jun 20;14(6):e1007457. doi: 10.1371/journal.pgen.1007457 (PMC6028147; doi:10.1371/journal.pgen.1007457)
Supplement: S1 Table — (PDF) [file pgen.1007457.s001.pdf]

S1 Table. CAPS markers used in mapping the *uv90* mutation

Marker Name	Restriction Enzyme	PCR Primers	SNP location (bp) on LG VI and PCR product location	SNP (OR > MV)	OR phenotype
LCF2-LCR2	HaeIII	TTTGAGCTCGGGATTTGCTG<br>GGGCGCCATCAATTTCACTT	1,900,315<br>1,900,164-1,900,640	C > T	cut
F11-R11	Taqα1	CGATACGATTCTTGGCATCAGC<br>GGTAACTATGTACATGCCCCGTG	2,075,827<br>2,075,729-2,076,173	G > A	cut
F9-R9*	Taqα1	AAGGTCGGGTTGTTAGACGA<br>GGCTGAGGGTGGATAGCTAGA	2,225,382<br>2,225,160-2,225,620	A > C	cut
F6-R6	EcoRI	TTCACGAGTGTCCCGGATGTG<br>GCATTGTCATCTCGTTGGTGC	***<br>2,493,864-2,495,098	***	cut
RCF5-RCR5	Taqα1	GCGAATTGGAAATCTCCAAGGG<br>ATCTCCCACGTAGCCTTGGCTT	3,102,537<br>3,102,391-3,102,921	G > A	cut
6-68-MspI**	MspI	ATGTCTTGGGTGTTTGGCAT<br>TCCTCAAGATCGTCACTCAGC	***<br>3,293,478-3,294,201	***	uncut
3317F-3317R	Taqα1	CTCGGACGACAAGTCACCAACA<br>CGTCCCTCTGATGCCCAAAGTA	3,317,544<br>3,317,357-3,317,859	A > G	cut
3368F-3368R	MseI	GAAATACCGGGCGCATTACCA<br>TGTTGATTGGCTGTCGGTGG	3,368,360<br>3,368,165-3,368,692	T > C	cut
3395F-3395R	Taqα1	TTGCTTGCTTTGTCTCGGACG<br>ACAGAGTTGGGTGTTCACGTGG	3,395,716<br>3,395,402-3,395,911	G > A	cut
3436F-3436R	Taqα1	GGACAGCTTCTCCGAGCGATTT<br>CGTTGACGGACTCTGAATGAGG	3,436,987<br>3,436,787-3,437,260	T > C	cut
F16-R16	HaeIII	ATCCAATCTCTTCCCGGTGTG<br>TCTTGGTTAGTGTAGCCGCACC	3,483,592<br>3,483,486-3,483,944	G > A	cut

* Marker and PCR primers from Lambreghts et al [70].
** Marker and PCR primers from Jin et al [34].
*** Exact SNP location not known.
