# Supplementary material for: A component of the TOR (Target Of Rapamycin) nutrient-sensing pathway plays a role in circadian rhythmicity in Neurospora crassa
Source: PLoS Genet. 2018 Jun 20;14(6):e1007457. doi: 10.1371/journal.pgen.1007457 (PMC6028147; doi:10.1371/journal.pgen.1007457)
Supplement: S2 Table — (PDF) [file pgen.1007457.s002.pdf]

S2 Table. Recombination frequencies between the *uv90* mutation and CAPS markers

Marker	Position on LG VI (bp)	Total progeny tested**	Total progeny recombinant	% recombin-ation
LCF2-LCR2	1,900,315	29	2	6.9
F11-R11	2,075,827	29	2	6.9
F9-R9	2,225,382	29	3	10.3
F6-R6	approx. 2,494,480*	29	3	10.3
centromere	2,811,000 – 3,060,000			
RCF5-RCR5	3,102,537	85	8	9.4
6-68-MspI	approx. 3,293,840*	85	3	3.5
F16-R16	3,483,592	85	8	9.4

* Exact SNP location is not known.

** Two different sets of progeny were tested on the left and right of the centromere.
