# Supplementary material for: A component of the TOR (Target Of Rapamycin) nutrient-sensing pathway plays a role in circadian rhythmicity in Neurospora crassa
Source: PLoS Genet. 2018 Jun 20;14(6):e1007457. doi: 10.1371/journal.pgen.1007457 (PMC6028147; doi:10.1371/journal.pgen.1007457)
Supplement: S3 Table — (PDF) [file pgen.1007457.s003.pdf]

S3 Table. ORF candidates for the *uv90* gene located on LG VI, from 3.293 to 3.369 Mbp

Locus	Gene Name	Location (bp, LG VI) and strand (+ or -)	FGSC Stock #*
NCU17127.7	hypothetical protein	3294468-3295683 +	**
NCU05962.7	hypothetical protein	3303139-3303378 -	17845
NCU05961.7	DUF572 domain-containing protein	3304910-3306530 +	11909
NCU05960.7	GPI mannosyltransferase 2 (gt-761)	3306630-3309661 -	**
NCU05959.7	vesicle transport V-SNARE protein VTI1	3311937-3313502 -	**
NCU05958.7	hypothetical protein	3315098-3318376 +	17842
NCU05957.7	hypothetical protein	3319864-3322467 +	13721
NCU05956.7	glycosylhydrolase family 2-2 (gh2-2)	3322517-3326426 -	11310
NCU05955.7	Cel74a (gh74-1)	3326807-3330420 +	13535
NCU05954.7	hypothetical protein	3332004-3334639 -	17841
NCU05953.7	hypothetical protein	3339700-3340998 -	**
NCU05952.7	hypothetical protein	3341267-3343308 -	17839
NCU05951.7	hypothetical protein	3344579-3346273 +	18085
NCU05950.7	hypothetical protein	3347867-3348756 +	18029
NCU05949.7	hypothetical protein	3349464-3350054 +	17838
NCU05948.7	unspecified product	3353034-3355088 -	18213
NCU05947.7	hypothetical protein	3355960-3356454 -	18028
NCU12130.7	hypothetical protein	3362030-3364815 +	**
NCU05946.7	hypothetical protein	3364883-3365627 -	17836
NCU05945.7	hypothetical protein	3366104-3367798 -	17835
NCU05944.7	hypothetical protein	3368035-3369538 +	13533

*Stock numbers of available knockout strains produced by the *Neurospora* Functional Genomics Project [65].

**Knockout strains of these genes were unavailable.
