# Supplementary material for: A component of the TOR (Target Of Rapamycin) nutrient-sensing pathway plays a role in circadian rhythmicity in Neurospora crassa
Source: PLoS Genet. 2018 Jun 20;14(6):e1007457. doi: 10.1371/journal.pgen.1007457 (PMC6028147; doi:10.1371/journal.pgen.1007457)
Supplement: S4 Table — (PDF) [file pgen.1007457.s004.pdf]

S4 Table. PCR primers used to analyze the UV90 mutation

	Primer Name	Sequence 5'→3'	Location on Linkage group VI and strand (+/-)
1	F6	GAC ATT GGC CGC AGA TGG AT	3,345,920- 939 (+)
2	F5	GGA ACC TCC GAA GAA AGG AAG G	3,346,404- 425 (+)
3	F4	CCG GAT GAC TTC ATC TTC CCT G	3,346,862- 883 (+)
4	F3	CTT GGA ACC GCG CAG TAA CAT	3,348,428- 448 (+)
5	F2	TAC TTC GAC GAC GTC TGG CTT C	3,347,890- 911 (+)
6	F1	CCG TGA CAC ATT TGT TGC TGC	3,347,371- 391 (+)
7	R1	TCC GGA ACA GCA TCA GGA AAG	3,348,966- 986 (-)
8	R2	CAT GGG TTT ATC ATG CGG GAC	3,348,468- 488 (-)
9	R3	CCG TCG CGA AGA GAT GAA ACT C	3,347,852- 873 (-)
10	R4	AAA TGG CTG TGG TCG CAT CTC	3,349,471- 491 (-)
11	R5	ATG AGG AAG CCG AGA GCA TCT C	3,349,938- 959 (-)
12	R6	CCG GTA TGA GCG TCG GAA TTT	3,350,451- 471 (-)
13	R7	GCC CGG ATG TTT GTT TCG TTC	3,350,986- 3,351,006 (-)
14	R8	TCA GTC TTT TTG GTC ACG GAC G	3,351,543- 564 (-)
15	R9	ACT CGC ACA TGC ATA CTC GCA	3,352,122- 142 (-)
16	UV90AFor	ACG TGG ACT GTT GCG TGA CAC	3,348,179- 199 (+)
17	UV90ARev	GTG TCA CGC AAC AGT CCA CGT	3,348,179- 199 (-)
18	UV90BFor	AAC TTT ACC GAT GCC GCT GC	3,348,687- 706 (+)
19	UV90BRev	GCA GCG GCA TCG GTA AAG TT	3,348,687- 706 (-)
20	FlipR1For	CTT TCC TGA TGC TGT TCC GGA	3,348,966- 986 (+)
21	FlipR4For	GAG ATG CGA CCA CAG CCA TTT	3,349,471- 491 (+)
22	FlipF5Rev	CCT TCC TTT CTT CGG AGG TTC C	3,346,425-404 (-)
23	FlipF4Rev	CAG GGA AGA TGA AGT CAT CCG G	3,346,883-862 (-)
24	FlipF1Rev	GCA GCA ACA AAT GTG TCA CGG	3,347,391-371 (-)
25	FlipR6For	AAA TTC CGA CGC TCA TAC CGG	3,350,451-471 (+)
26	FlipR7For	GAA CGA AAC AAA CAT CCG GGC	3,350,986- 3,351,006 (+)
27	FlipR8For	CGT CCG TGA CCA AAA AGA CTG A	3,351,543-564 (+)
28	17F1	ACG CTT CTT TCC TTA TCC CAC CCA C	3,344,879-903 (+)
29	17F2	AGG TAG AAG TTG GAA AGG TGC GCT G	3,343,693–717 (+)
30	17F3	TCG GTC TTG ATC GCT GGT TCT TAC G	3,342,614-638 (+)
31	17F4	GTA CGC CGT GTA TTT GTG TGT CTG G	3,341,477-501 (+)
32	17F5	TCC TCC ATC ACC AAC GCA CAA TGA C	3,339,947-971 (+)
33	17F6	AGC CCG CTA ACG CCA ATC TTA TAC	3,338,357-380 (+)
34	17F7	AAT CTA AAG GAG GAG GAC CCG GAA G	3,337,024-048 (+)
35	17F8	CCG GCG CAA ACC ATC GAA ATT AAG	3,335,528-551 (+)
36	17F9	CTT GTG AAT GGT TTG TGG CGG TTC C	3,334,358-382 (+)
37	17F10	TCT CCG TCT CCG TAT TGG GTA CAT C	3,333,239-263 (+)
38	17F11	CAT TGC CAT TCC CAA CAA CAG GTG	3,332,263-286 (+)

39	17F12	GCC CAA CAA ACA ACG AAC AAC GAT C	3,331,005-029 (+)
40	17F13	CAA CGG CAA CAA GCT GTA CGC TAG	3,329,680-703 (+)
41	17F14	AGT TCC GAC AGA GGT GCT ACA TGG	3,327,929-952 (+)
42	17F15	AAC AGC ACT GAC ATC AGC AAG CAA G	3,326,728-752 (+)
43	17F16	GCT TGG CAT CCT TGT ACT CGT TGT C	3,325,372-396 (+)
44	17F17	TCT CAA TCC ATT CGG GCT CAT CGT C	3,324,110-134 (+)
45	17F18	ACT CGT ATT CTC TCC ATT GGG CTC G	3,323,122-146 (+)
46	17F19	TGG TGT GAT GGA AAT GTA CGC GCT G	3,321,779-803 (+)
47	17F20	CTG GAA CCC ATT TCT CGC ACA CAT C	3,320,481-505 (+)
48	17F21	TTT GGT TGT CTG GTT GGC GTT ATC C	3,319,264-288 (+)
49	17F22	GTG AGG CCA ACG ACT GAG AAA GTA G	3,317,916-940 (+)
50	17F23	ACA GTG ACG AGA CCT TTG AGA GGA G	3,316,708-732 (+)
51	17F24	CCT TGC TTC ACC ACG TTC TTA GCT C	3,315,419-443 (+)
52	17F25	TAC ACT CTC TCT GTT GCT CGT CGT C	3,314,341-365 (+)
53	17F26	TGT TGC TAT CGG ATG TGG AGG TTG G	3,313,207-231 (+)
54	17F27	CTC CAT CGG TAC AGT CCA AAC CAT C	3,312,220-244 (+)
55	17F28	TGA AGA CTG CCA CCA CGT TGA TAC	3,310,921-944 (+)
56	17F29	ATA CAA GAC ACG ACC ACA CGC CAA G	3,309,813-837 (+)
57	17F30	GGC AAG TGT GGA TTG GTG ATG TGA G	3,308,466-490 (+)
58	17R1	CAA CGG AGA GAG AGG GTG AAT GGA A	3,351,689-665 (-)
59	17R2	CGA CAG GCA AAG GTA AGA AAG GGA G	3,352,758-734 (-)
60	17R3	GAC AAT TTC CAC CGT GCT ACC ACC A	3,353,926-902 (-)
61	17R4	GAG GGC CGG AAA GAA GTC AAA CCA A	3,355,140-116 (-)
62	17R5	CCA CCC TCA CCG TTC AGA TAC TTC T	3,356,189-165 (-)
63	17R6	CCT TCC ACT CAA CAG TTC GTC CGT T	3357,753-729 (-)
64	17R7	CAT ATT CCT ACT TGA CGC CGC TCA G	3,358,854-830 (-)
65	17R8	CAA GTA CAC CAC TCC CAC CAC ACT	3,360,081-058 (-)
66	17R9	CTC TGT CCT TCT CCA GCC TTC TTG A	3,361,047-023 (-)
67	17R10	CAA GAC GGG AGT GGA AGA GTC AAA G	3,362,202-178 (-)
68	17R11	CAT GCC ATC CCA CTC CCT TGA AGA T	3,363,468-444 (-)
69	17R12	CTC GGT CAA ATG GTG GAT GTG TTG C	3,364,946-922 (-)
70	17R13	CCA AGT CCC ATG ACG AAG AGC AAT C	3,366,785-761 (-)
71	17R14	CGT CTT GCC TAT TCC CAC TCC TTT C	3,368,511-535 (-)
72	17R6FlipFor	AAC GGA CGA ACT GTT GAG TGG AAG G	3357,729-753 (+)
73	17R7FlipFor	CTG AGC GGC GTC AAG TAG GAA TAT G	3,358,830-854 (+)
74	17R8FlipFor	AGT GTG GTG GGA GTG GTG TAC TTG	3,360,058-081 (+)
75	17R9FlipFor	TCA AGA AGG CTG GAG AAG GAC AGA G	3,361,023-047 (+)
76	17R10FlipFor	CTT TGA CTC TTC CAC TCC CGT CTT G	3,362,178-202 (+)
77	17R11FlipFor	ATC TTC AAG GGA GTG GGA TGG CAT G	3,363,444-468 (+)
78	17R12FlipFor	GCA ACA CAT CCA CCA TTT GAC CGA G	3,364,922-946 (+)
79	17R13FlipFor	GAT TGC TCT TCG TCA TGG GAC TTG G	3,366,761-785 (+)
80	17F6FlipRev	GTA TAA GAT TGG CGT TAG CGG GCT	3,338,380-357 (-)
81	17F7FlipRev	CTT CCG GGT CCT CCT CCT TTA GAT T	3,337,048-024 (-)
82	17F8FlipRev	CTT AAT TTC GAT GGT TTG CGC CGG	3,335,551-528 (-)
83	17F9FlipRev	GGA ACC GCC ACA AAC CAT TCA CAA G	3,334,382-358 (-)

84	17F10FlipRev	TCT CCG TCT CCG TAT TGG GTA CAT C	3,333,263-239 (-)
85	17F11FlipRev	CAC CTG TTG TTG GGA ATG GCA ATG	3,332,286-263 (-)
86	17F12FlipRev	GAT CGT TGT TCG TTG TTT GTT GGG C	3,331,029-005 (-)
87	17F13FlipRev	CTA GCG TAC AGC TTG TTG CCG TTG	3,329,703-680 (-)
88	17F14FlipRev	CCA TGT AGC ACC TCT GTC GGA ACT	3,327,952-929 (-)
89	17F15FlipRev	CTT GCT TGC TGA TGT CAG TGC TGT T	3,326,752-728 (-)
90	17F16FlipRev	GAC AAC GAG TAC AAG GAT GCC AAG C	3,325,396-372 (-)
91	17F17FlipRev	GAC GAT GAG CCC GAA TGG ATT GAG A	3,324,134-110 (-)
92	17F18FlipRev	CGA GCC CAA TGG AGA GAA TAC GAG T	3,323,146-122 (-)
93	17F19FlipRev	CAG CGC GTA CAT TTC CAT CAC ACC A	3,321,803-779 (-)
94	17F20FlipRev	GAT GTG TGC GAG AAA TGG GTT CCA G	3,320,505-481 (-)
95	17F21FlipRev	GGA TAA CGC CAA CCA GAC AAC CAA A	3,319,288-264 (-)
96	17F22FlipRev	CTA CTT TCT CAG TCG TTG GCC TCA C	3,317,940-916 (-)
97	17F23FlipRev	CTC CTC TCA AAG GTC TCG TCA CTG T	3,316,732-708 (-)
98	17F24FlipRev	GAG CTA AGA ACG TGG TGA AGC AAG G	3,315,443-419 (-)
99	17F25FlipRev	GAC GAC GAG CAA CAG AGA GAG TGT A	3,314,365-341 (-)
100	17F26FlipRev	CCA ACC TCC ACA TCC GAT AGC AAC A	3,313,231-207 (-)
101	17F27FlipRev	GAT GGT TTG GAC TGT ACC GAT GGA G	3,312,244-220 (-)
102	17F28FlipRev	GTA TCA ACG TGG TGG CAG TCT TCA	3,310,944-921 (-)
103	17F29FlipRev	CTT GGC GTG TGG TCG TGT CTT GTA T	3,309,837-813 (-)
104	18SeqF1	GAC CCT GAC ACG AAA TAA ACG TGC	3,320,837-860 (+)
105	18SeqR1	CTC TAA TGC CGC TAA TCC ACG GAT	3,357,216-193 (-)
106	18SeqF2	TCC CTA TCC CAC TCG AAC ATA ATG C	3,321,025-049 (+)
107	18SeqR2	GAC CGG CTT CTA CTC TCG TTC AA	3,356,726-704 (-)
108	18SeqR3	CAC ACG CAA AGA GAG GAA GGA GAC A	3,356,267-243 (-)

Primers #1-15 ordered from Invitrogen. All other primers ordered from Integrated DNA Technologies (IDT).
