# Supplementary material for: A component of the TOR (Target Of Rapamycin) nutrient-sensing pathway plays a role in circadian rhythmicity in Neurospora crassa
Source: PLoS Genet. 2018 Jun 20;14(6):e1007457. doi: 10.1371/journal.pgen.1007457 (PMC6028147; doi:10.1371/journal.pgen.1007457)
Supplement: S5 Table — (PDF) [file pgen.1007457.s005.pdf]

S5 Table. Top ten sequences similar to NCU05950*

NCBI/Gene bank sequence reference number	Protein function and name	Organism	E value
XP_009854653.1	Hypothetical protein NEUTE1DRAFT_69638	*Neurospora tetrasperma*	5e-114
XP_003349138.1	Hypothetical protein SMAC_06974	*Sordaria macrospora*	1e-103
XP_003650216.1	Hypothetical protein THITE_2063314	*Thielavia terrestris*	2e-60
KXX83062.1	Hypothetical protein MMYC01_200469	*Madurella mycetomatis*	6e-59
XP_001227334.1	Hypothetical protein CHGG_09407	*Chaetomium globosum*	8e-57
XP_006696736.1	Hypothetical protein CTHT_0064310	*Chaetomium thermophilum*	2e-56
XP_003663911.1	Hypothetical protein MYCTH_2315477	*Myceliophthora thermophile*	1e-55
XP_001906340.1	Hypothetical protein	*Podospora anserina*	3e-48
XP_008095402.1	Hypothetical protein GLRG_06526	*Colletotrichum graminicola*	4e-39
KZL63082.1	Hypothetical protein CI238_05869	*Colletotrichum incanum*	2e-38

*The NCU05950 protein sequence was used as the query for a protein BLAST search in the NCBI database of non-redundant coding sequences from GenBank.
