# Supplementary material for: A component of the TOR (Target Of Rapamycin) nutrient-sensing pathway plays a role in circadian rhythmicity in Neurospora crassa
Source: PLoS Genet. 2018 Jun 20;14(6):e1007457. doi: 10.1371/journal.pgen.1007457 (PMC6028147; doi:10.1371/journal.pgen.1007457)
Supplement: S6 Table — (PDF) [file pgen.1007457.s006.pdf]

S6 Table. Periods and growth rates of NCU05950 mutants and transformants

		+ choline		- choline	
Genotype	Number of Isolates	period (h)	growth rate (mm/h)	period (h)	growth rate (mm/h)
control	1	21.9 ± 0.2 (12)	1.38 ± 0.04 (12)	47.3 ± 1.8 (16)	0.54 ± 0.01 (16)
frq^{null}	1	N.R.	1.46 ± 0.01 (4)	56.5 ± 4.3 (8)	0.50 ± 0.01 (8)
uv90	1	22.1 ± 0.2 (12)	1.20 ± 0.02 (12)	N.R.	0.52 ± 0.01 (16)
uv90 frq^{null}	1	N.R.	1.28 ± 0.03 (12)	N.R.	0.57 ± 0.01 (16)
NCU05950^{KO}	1	23.1 ± 0.6 (8)	1.19 ± 0.02 (8)	N.R.	0.50 ± 0.02 (8)
NCU05950^{KO} *frq*^{null}	1	N.R.	1.18 ± 0.01 (8)	N.R.	0.51 ± 0.01 (8)
uv90 NCU05950^{+}	3	21.2 ± 0.1 (12)	1.39 ± 0.01 (12)	57.6 ± 3.0 (24)	0.48 ± 0.01 (24)
uv90 frq^{null} NCU05950^{+}	3	N.R.	1.39 ± 0.02 (12)	61.2 ± 2.9 (23)	0.49 ± 0.01 (24)

Genotype abbreviations as for Fig. 2. All strains are also *csp-1; ras*^{bd} *chol-1*.
Data from transformants pooled from several independent isolates with the indicated genotype.
Strains were grown with (+) or without (-) 100 µM choline in the medium.
Data reported as mean ± S.E.M. (N)
N.R.: Not rhythmic
