# Supplementary material for: A component of the TOR (Target Of Rapamycin) nutrient-sensing pathway plays a role in circadian rhythmicity in Neurospora crassa
Source: PLoS Genet. 2018 Jun 20;14(6):e1007457. doi: 10.1371/journal.pgen.1007457 (PMC6028147; doi:10.1371/journal.pgen.1007457)
Supplement: S7 Table — (PDF) [file pgen.1007457.s007.pdf]

**S7 Table. Periods and growth rates of NCU05950-GFP and –FLAG fusion proteins**

| | | + choline | | - choline | |
|---|---|---|---|---|---|
| Genotype | Number of Isolates | period (h) | growth rate (mm/h) | period (h) | growth rate (mm/h) |
| control | 1 | 21.3 ± 0.1 (10) | 1.34 ± 0.02 (10) | 51.8 ± 1.7 (10) | 0.49 ± 0.01 (10) |
| NCU05950$^{KO}$ | 1 | 22.3 ± 0.3 (10) | 1.16 ± 0.04 (10) | N.R. | 0.49 ± 0.01 (10) |
| pCCG::NCU05950:: GFP | 6 | 21.5 ± 0.1 (18) | 1.24 ± 0.01 (18) | 57.7 ± 2.0 (18) | 0.47 ± 0.01 (18) |
| NCU05950:: GFP | 4 | 21.4 ± 0.1 (12) | 1.21 ± 0.01 (12) | 54.4 ± 3.0 (11) | 0.46 ± 0.01 (12) |
| NCU05950:: FLAG | 4 | 22.0 ± 0.1 (16) | 1.28 ± 0.01 (16) | 45.6 ± 1.5 (14) | 0.51 ± 0.01 (14) |
| Δ1-NCU05950:: FLAG | 2 | 21.9 ± 0.2 (8) | 1.27 ± 0.003 (8) | 47.7 ± 2.8 (8) | 0.49 ± 0.01 (8) |
| Δ7-NCU05950:: FLAG | 2 | 23.4 ± 0.3 (8) | 1.17 ± 0.06 (8) | N.R. | 0.50 ± 0.01 (8) |
| Δ10-NCU05950:: FLAG | 2 | 24.2 ± 0.3 (8) | 1.04 ± 0.01 (8) | N.R. | 0.50 ± 0.01 (8) |

Genotype abbreviations as for Fig. 6. All strains are also *csp-1; ras$^{bd}$ chol-1*.
Data from transformants pooled from several independent isolates with the indicated genotype.
Strains were grown with (+) or without (-) 100 μM choline in the medium.
Data reported as mean ± S.E.M. (N)
N.R.: Not rhythmic
