# Supplementary material for: A component of the TOR (Target Of Rapamycin) nutrient-sensing pathway plays a role in circadian rhythmicity in Neurospora crassa
Source: PLoS Genet. 2018 Jun 20;14(6):e1007457. doi: 10.1371/journal.pgen.1007457 (PMC6028147; doi:10.1371/journal.pgen.1007457)
Supplement: S8 Table — (PDF) [file pgen.1007457.s008.pdf]

S8 Table. Periods and growth rates of GFP-tagged N-terminal deletion strains

	+ choline		- choline	
Genotype	period (h)	growth rate (mm/h)	period (h)	growth rate (mm/h)
control	21.3 ± 0.08	1.55 ± 0.01	46.3 ± 4.5	0.53 ± 0.03
$NCU05950^{KO}$	22.4 ± 0.01	1.36 ± 0.01	N.R.	0.53 ± 0.03
NCU05950::GFP	21.2 ± 0.04	1.46 ± 0.003	38.8 ± 1.8	0.54 ± 0.01
Δ1-NCU05950::GFP	21.6 ± 0.14	1.47 ± 0.01	43.5 ± 2.0	0.52 ± 0.003
Δ7-NCU05950::GFP	22.0 ± 0.16	1.41 ± 0.003	N.R.	0.56 ± 0.01
Δ10-NCU05950::GFP	22.1 ± 0.13	1.48 ± 0.01	N.R.	0.56 ± 0.01

Genotype abbreviations as for S4 Fig. All strains are also *csp-1; ras^{bd} chol-1*.
Strains were grown with (+) or without (-) 100 µM choline in the medium.
Data are from one isolate of each genotype and three replicate race tubes per condition.
Data are reported as mean ± S.E.M. (N=3)
N.R.: Not rhythmic
