# Supplementary material for: A component of the TOR (Target Of Rapamycin) nutrient-sensing pathway plays a role in circadian rhythmicity in Neurospora crassa
Source: PLoS Genet. 2018 Jun 20;14(6):e1007457. doi: 10.1371/journal.pgen.1007457 (PMC6028147; doi:10.1371/journal.pgen.1007457)
Supplement: S1 Fig — Representative gels of PCR products derived from the regions around the uv90 deletion. Top row: Primer pairs on either side of the deletion breakpoints showing absence or presence of products in the uv90 mutant. Bottom row: long-range PCR across the breakpoint showing presence of shorter products in the uv90 mutant. Two strains of each genotype (uv90 wild-type “control” and uv90 mutant) are shown. Genotypes are: control 1, csp-1; chol-1 rasbd; control 2, csp-1; chol-1 rasbd; frq10; uv90 mutant 1, csp-1; chol-1 rasbd; uv90; uv90 mutant 2, csp-1; chol-1 rasbd; uv90; frq10. PCR primers are listed in S4 Table and are mapped in S2 Fig. (PDF) [file pgen.1007457.s009.pdf]

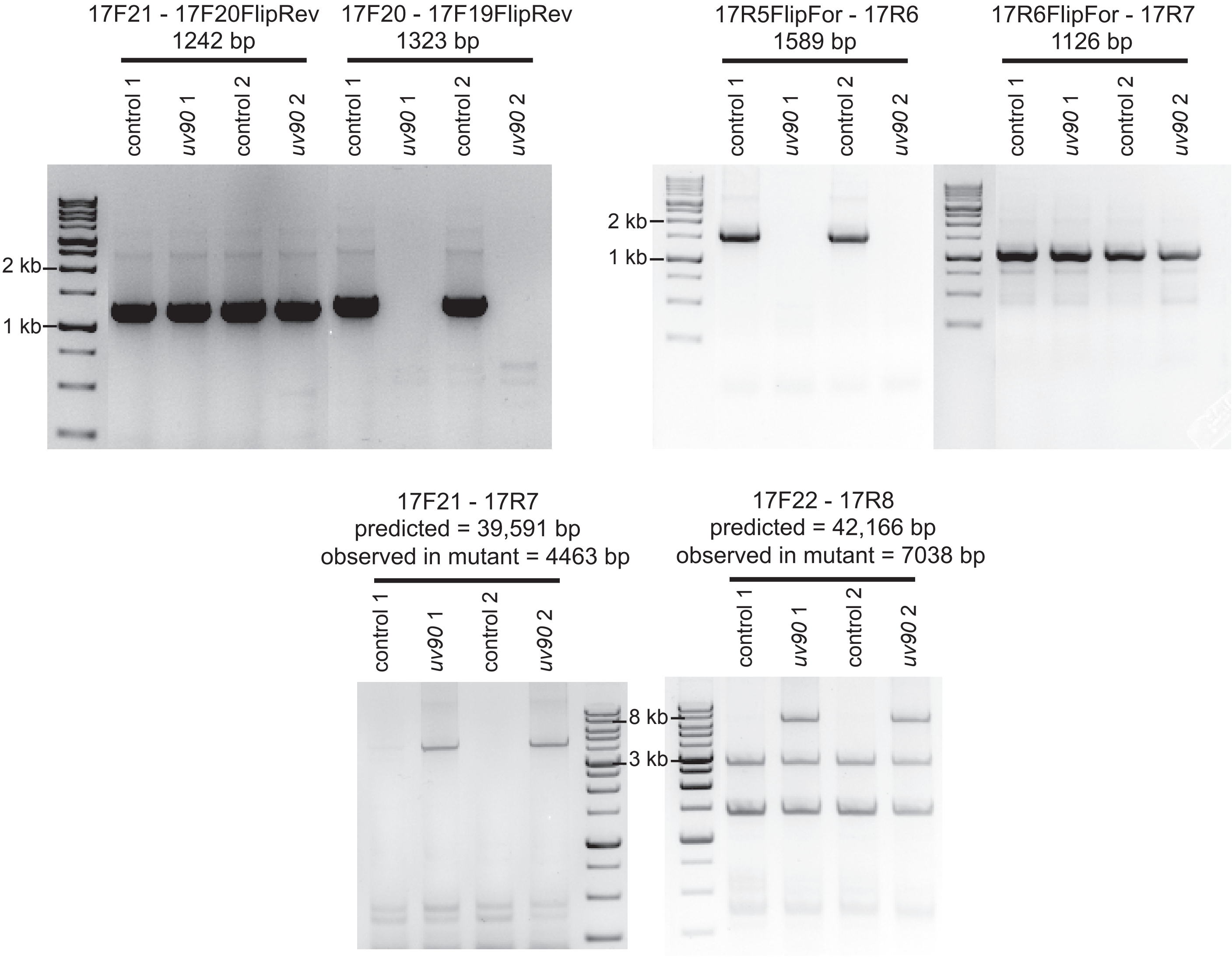
17F21 - 17F20FlipRev
1242 bp
17F20 - 17F19FlipRev
1323 bp
17R5FlipFor - 17R6
1589 bp
17R6FlipFor - 17R7
1126 bp
control 1
uv90 1
control 2
uv90 2
2 kb
1 kb
17F21 - 17R7
predicted = 39,591 bp
observed in mutant = 4463 bp
17F22 - 17R8
predicted = 42,166 bp
observed in mutant = 7038 bp
8 kb
3 kb
