# Supplementary material for: A component of the TOR (Target Of Rapamycin) nutrient-sensing pathway plays a role in circadian rhythmicity in Neurospora crassa
Source: PLoS Genet. 2018 Jun 20;14(6):e1007457. doi: 10.1371/journal.pgen.1007457 (PMC6028147; doi:10.1371/journal.pgen.1007457)
Supplement: S2 Fig — Nucleotide positions on chromosome VI and gene sizes are shown for genes. Marks are at 1 kb intervals. PCR primers shown above the chromosome are listed in S4 Table. (PDF) [file pgen.1007457.s010.pdf]

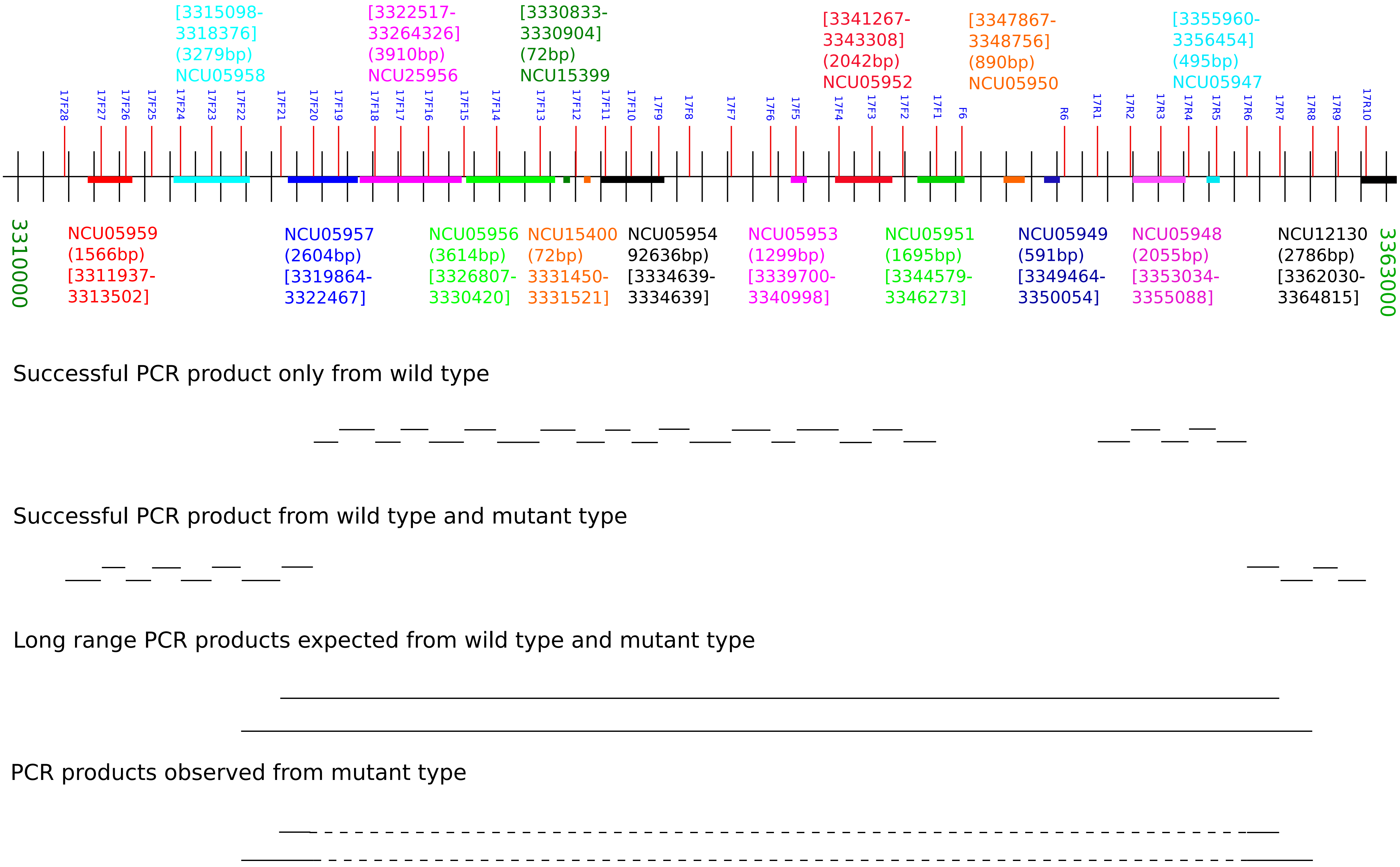
[3315098-3318376] (3279bp) NCU05958
[3322517-33264326] (3910bp) NCU25956
[3330833-3330904] (72bp) NCU15399
[3341267-3343308] (2042bp) NCU05952
[3347867-3348756] (890bp) NCU05950
[3355960-3356454] (495bp) NCU05947
17F28 17F27 17F26 17F25 17F24 17F23 17F22 17F21 17F20 17F19 17F18 17F17 17F16 17F15 17F14 17F13 17F12 17F11 17F10 17F9 17F8 17F7 17F6 17F5 17F4 17F3 17F2 17F1 F6
R6 17R1 17R2 17R3 17R4 17R5 17R6 17R7 17R8 17R9 17R10
3310000
NCU05959 (1566bp) [3311937-3313502]
NCU05957 (2604bp) [3319864-3322467]
NCU05956 (3614bp) [3326807-3330420]
NCU15400 (72bp) 3331450-3331521]
NCU05954 92636bp) [3334639-3334639]
NCU05953 (1299bp) [3339700-3340998]
NCU05951 (1695bp) [3344579-3346273]
NCU05949 (591bp) [3349464-3350054]
NCU05948 (2055bp) [3353034-3355088]
NCU12130 (2786bp) [3362030-3364815]
3363000
Successful PCR product only from wild type
Successful PCR product from wild type and mutant type
Long range PCR products expected from wild type and mutant type
PCR products observed from mutant type
