# Supplementary material for: A component of the TOR (Target Of Rapamycin) nutrient-sensing pathway plays a role in circadian rhythmicity in Neurospora crassa
Source: PLoS Genet. 2018 Jun 20;14(6):e1007457. doi: 10.1371/journal.pgen.1007457 (PMC6028147; doi:10.1371/journal.pgen.1007457)
Supplement: S3 Fig — (PDF) [file pgen.1007457.s011.pdf]

N.crassa* vs. *S. cerevisiae

```
N    1 MGNFCSTCFGGRRSDDYDEEDEAQHLFDENNMHYGS-------FDQQHMM  43
       ||...|.|     .:...||:||  |..|....|||       :|.:.|.
S    1 MGAVLSCC-----RNHSGEENEA--LLREQQAGYGSQGNANDEYDAEQMR  43

N   44 NQEDPQETEREIAA----LQGVVERTSNNMVDI-----YDMVPHDKPMQD  84
       .:|  .|.|:::.|    |:.:|..|::.::||     ..:|.....:|:
S   44 LKE--HEHEQKLLAREQELRDIVANTNDKLIDISMINNSGIVIQGTDLQE  91

N   85 APAPYGFANQRYNALLSKLSTHDDMAAVARVDWGTPEDDSMEMLRKASLP 134
       |              |.|....:.             .||.|..|.|
S   92 A--------------LDKRQQEEG-------------GDSREDERSA--- 111

N  135 TIPIKAEGGEALVGNFTDAAAAMR-------------------------- 158
              |.:.|.|:...::.:.:
S  112 -------GDDNLSGHSVPSSGSAQATTHQTAPRTNTFTLLTSPDSAKISK 154

N  159 ------------------------------ 158

S  155 EQLKKLHSNILNEIFSQSQVNKPGPLTVPF 184
```

Identity: 37/230 (16.1%)
Similarity: 61/230 (26.5%)
Gaps: 118/230 (51.3%)
Score: 49.5

N.crassa* vs. *H. sapiens

```
N   1 MGNFCSTCFGGRRSDDYDEEDEAQHLFDENN----MHYGSFDQQHMMNQE 46
      ||  |  |:.....|...:.:|.:.|.|.::    ...|:....|.:
H   1 MG--C--CYSSENEDSDQDREERKLLLDPSSPPTKALNGAEPNYHSL--- 43

N  47 DPQETEREIAALQGVVERTSNNMVDI-----YDMVPHDKPMQDAPAPYGF 91
       |.....|.|.|..::.:|::|::|:     ..|..|:         |..
H  44 -PSARTDEQALLSSILAKTASNIIDVSAADSQGMEQHE---------YMD 83

N  92 ANQRYN---ALLSKLSTHDDMAAVARVDWGTPEDDSMEMLRKASLPTIP- 137
      ..::|:   |:||...||          |             ..||.:|
H  84 RARQYSTRLAVLSSSLTH----------W-------------KKLPPLPS 110

N 138 IKAEGGEALVGN---FTDAAAAMR-------------------------- 158
      :.::..:.|...   |:|.....|
H 111 LTSQPHQVLASEPIPFSDLQQVSRIAAYAYSALSQIRVDAKEELVVQFGI 160

N 159 - 158

H 161 P 161
```

Identity: 34/201 (16.9%)
Similarity: 61/201 (30.3%)
Gaps: 83/201 (41.3%)
Score: 55.5

KEY:
Conserved LAMTOR domains: blue
Potential myristoylation sites: yellow
Potential palmitoylation sites: green
Potential lysosomal/vacuolar localization signals: **underlined bold**
| Identity
.: Similarities
