# Supplementary figures and images for: A component of the TOR (Target Of Rapamycin) nutrient-sensing pathway plays a role in circadian rhythmicity in Neurospora crassa
Source: PLoS Genet. 2018 Jun 20;14(6):e1007457. doi: 10.1371/journal.pgen.1007457 (PMC6028147; doi:10.1371/journal.pgen.1007457)

+ choline

- choline

control

NCU05950<sup>KO</sup>

NCU05950::GFP

$\Delta$ 1-NCU05950::GFP

$\Delta$ 7-NCU05950::GFP

$\Delta$ 10-NCU05950::GFP

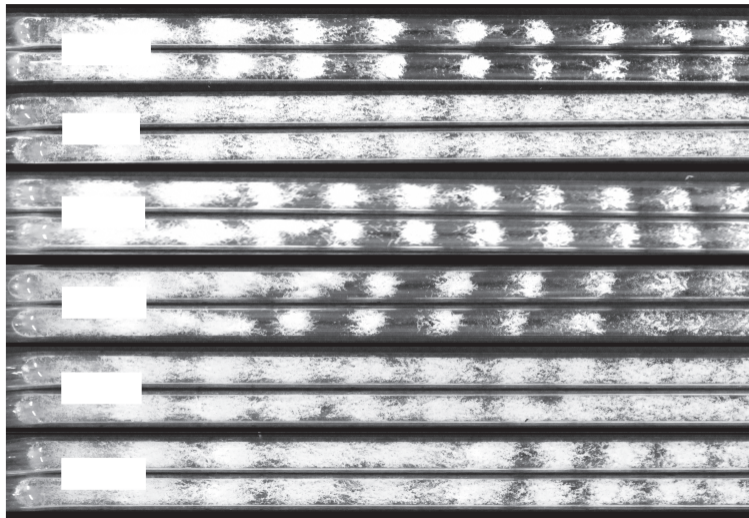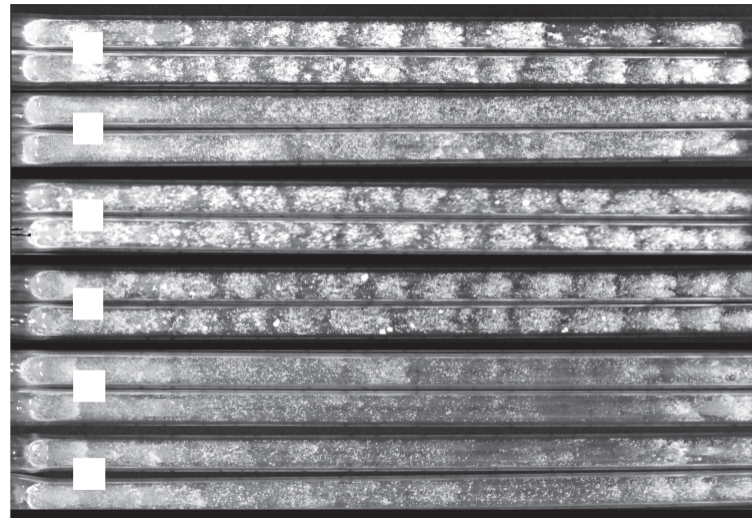

Supplement: S4 Fig — All strains, including control, have the csp-1; rasbd chol-1 genetic background. All strains except the control also carry the NCU05950 deletion allele (NCU05950KO). GFP-tagged fusion genes were inserted at the his-3 locus of a NCU05950KO strain and were expressed from the native NCU05950 promoter. Δ1, Δ7 and Δ10: The GFP fusion protein of NCU05950 was modified by deleting either 1, 7 or 10 amino acids after the initial methionine at the N-terminus. Strains were grown with (+, left panel) or without (-, right panel) 100 μM choline in the medium. Two representative replicate tubes for each condition are shown. Growth is from left to right. White bars indicate average growth in 24 hours. Periods and growth rates are reported in S8 Table. (PDF) [file pgen.1007457.s012.pdf]

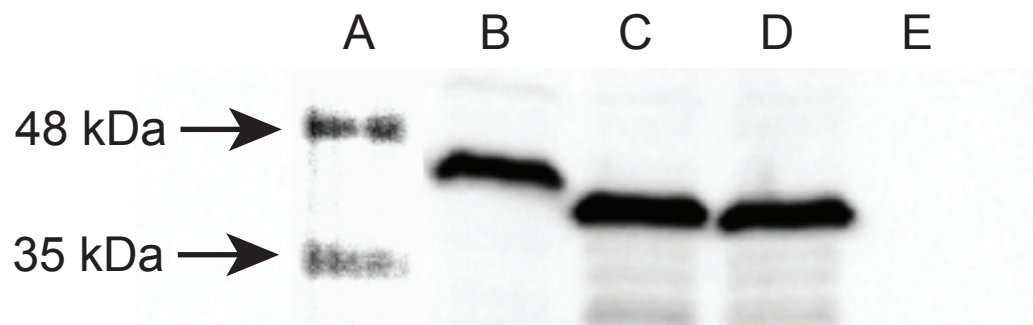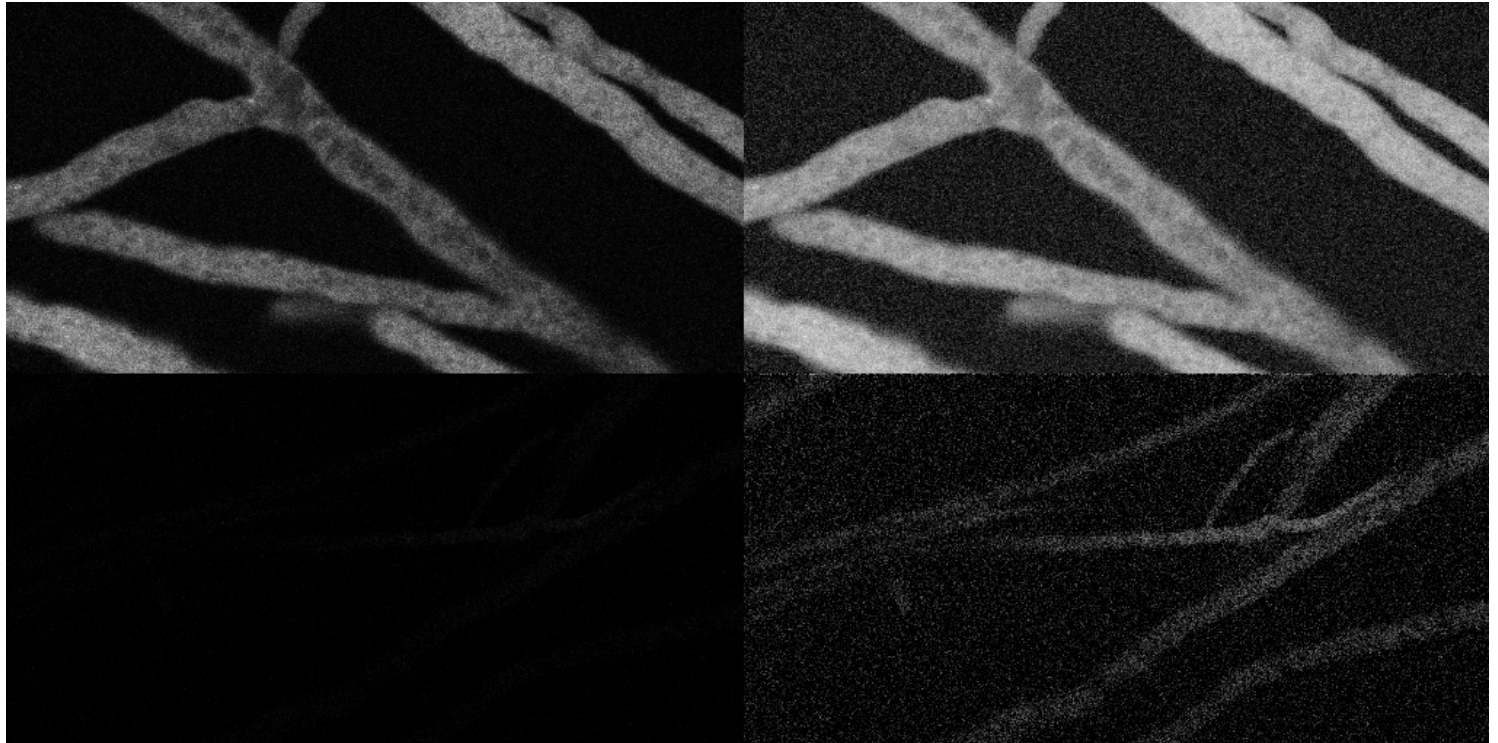

Supplement: S5 Fig — Top panel: Western blot of GFP-tagged proteins expressed from the ccg-1 promoter. The expected size of the tagged full-length protein is 44.6 kDa. Strains are described in Fig 7B. Lane A: molecular weight markers. Lane B: full-length GFP-tagged protein. Lane C: Δ7 deletion. Lane D: Δ10 deletion. Lane E: untagged control strain. Bottom panel: Confocal microscopy of GFP-tagged protein expressed from the native promoter. Strains and conditions as for Fig 7C. Top row: N-terminal Δ10 deletion. Bottom row: untagged control strain, showing autofluorescence. Left column: Images taken with the same microscope brightness and contrast settings. Right column: Images with levels adjusted in Photoshop to show autofluorescence of control strain. (PDF) [file pgen.1007457.s013.pdf]

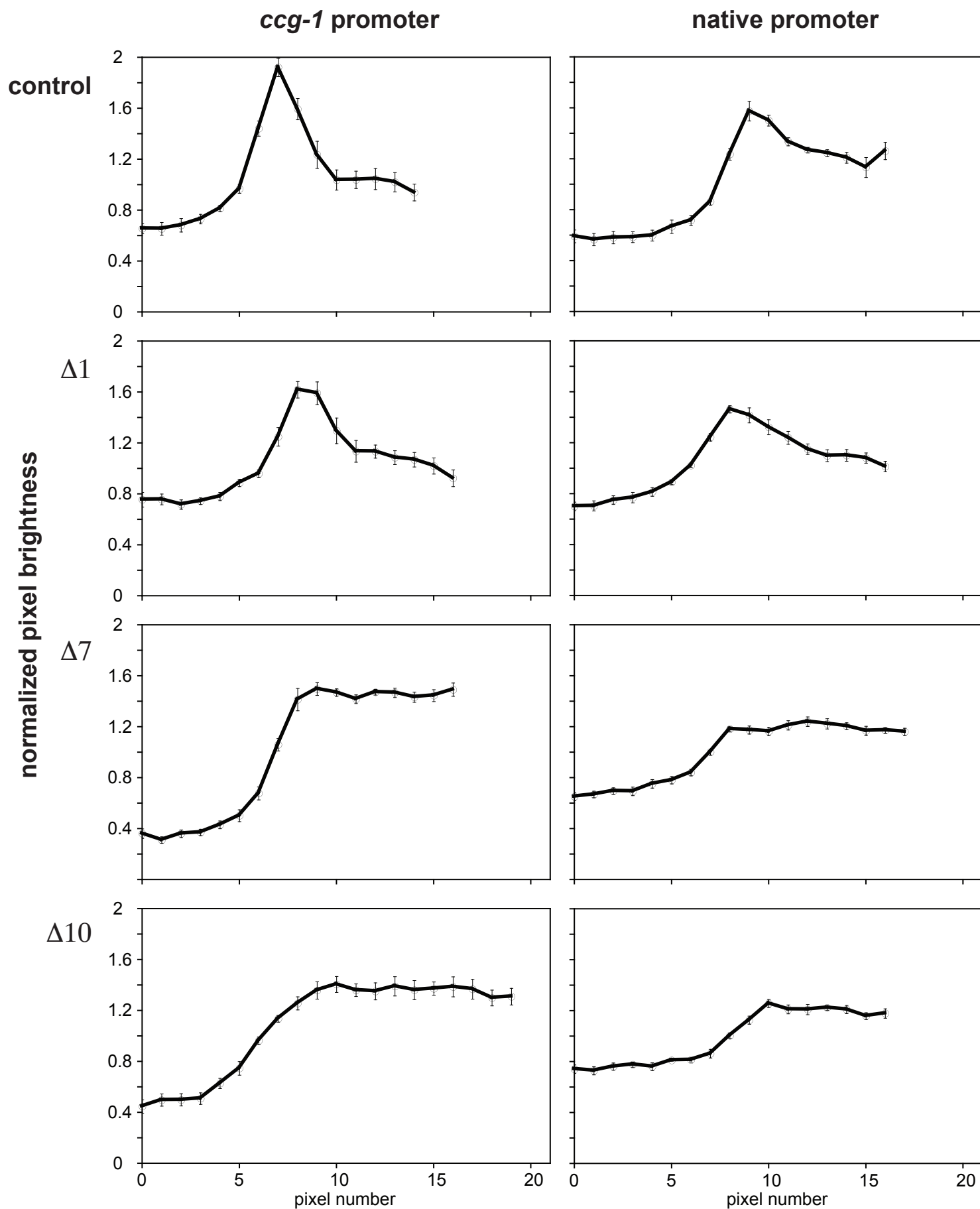

Supplement: S6 Fig — Images related to Fig 7 were analyzed by quantitating pixel brightness across transects drawn from the interior of vacuoles across the vacuolar membrane into the cytosol. Pixel number zero is in the interior of the vacuole. Scale: 10 pixels = 4 μm. Six vacuoles per strain were quantitated. Error bars are mean ± SEM (N = 6). Left column: NCU05950-GFP driven by the ccg-1 promoter. Right column: NCU05950-GFP driven by the native promoter. Top row: full-length NCU05950 gene fused to GFP (control). Rows 2, 3 and 4: N-terminal deletions of NCU05950 fused to GFP, deleting 1, 7 or 10 amino acids. (PDF) [file pgen.1007457.s014.pdf]

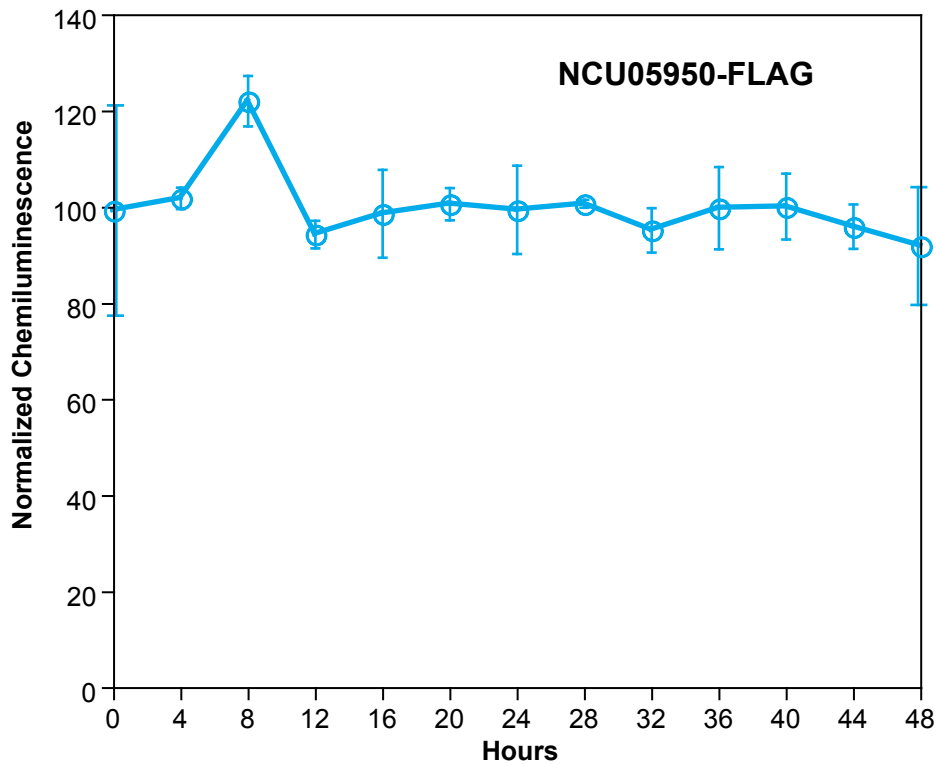

Supplement: S7 Fig — Cultures were grown on top of cellophane overlaid on solid agar medium. Samples were collected every 4 hours, processed for immunoblotting, and analysed as for Fig 8. Data points are mean ± S.E.M. of three independent experiments. (PDF) [file pgen.1007457.s015.pdf]

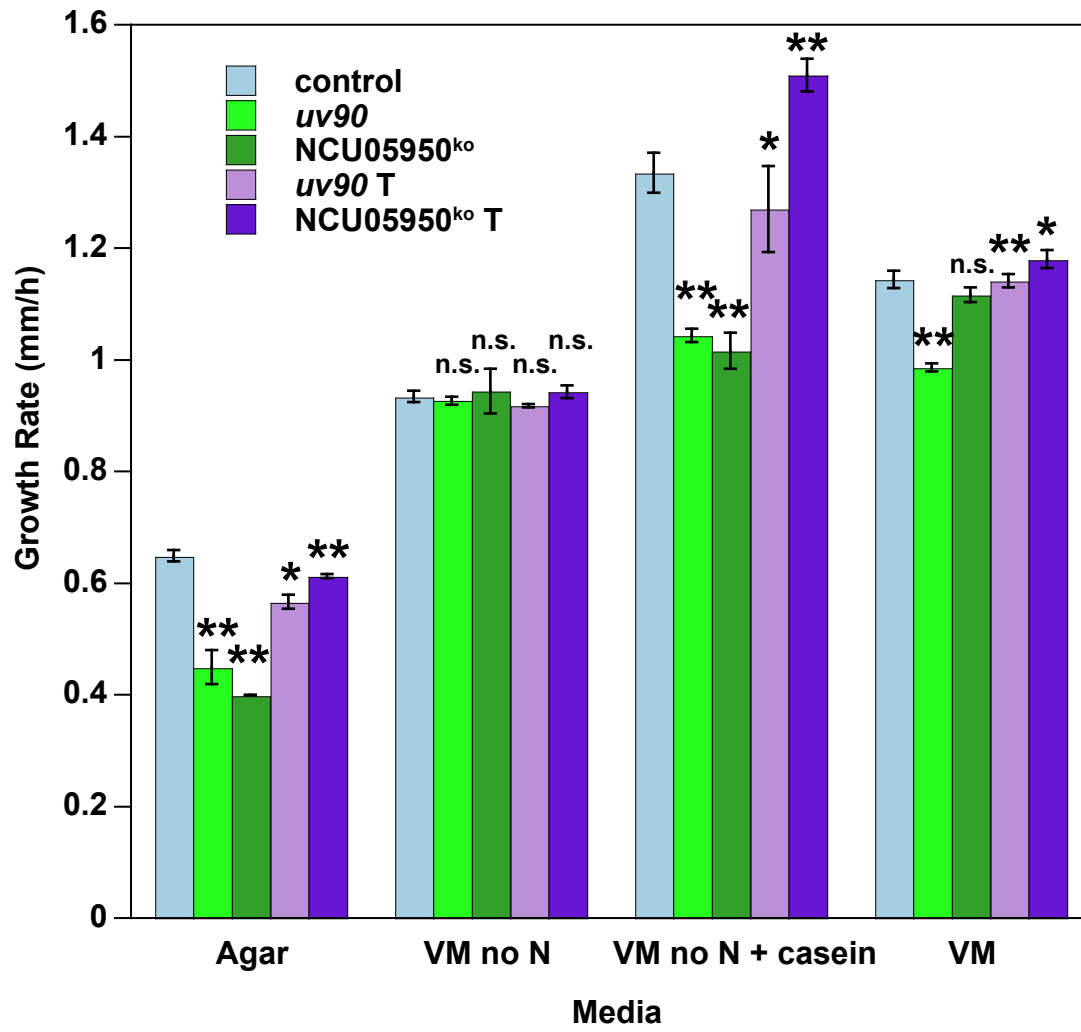

Supplement: S8 Fig — Strains were grown on race tubes containing various growth media as described in Fig 9. All strains carried the csp-1; rasbd chol-1 background and were grown with 100 μM choline added to the media. uv90 strains are the original uv90 mutant. NCU05950ko strains are the deletion mutant. Strains marked T (for transformant) also carried a wild-type copy of NCU05950 inserted at the his-3 locus. Values are the mean of three replicate race tubes and error bars are ± S.E.M. Stars (*) indicate statistically significant difference between mutant and control, or between transformant and corresponding mutant; *, p < 0.05; **, p < 0.01; n.s., not significantly different. (PDF) [file pgen.1007457.s016.pdf]
